# Novel Ethanol- and 5-Hydroxymethyl Furfural-Stimulated β-Glucosidase Retrieved From a Brazilian Secondary Atlantic Forest Soil Metagenome

**DOI:** 10.3389/fmicb.2018.02556

**Published:** 2018-10-29

**Authors:** Luana de Fátima Alves, Luana Parras Meleiro, Roberto N. Silva, Cauã Antunes Westmann, María-Eugenia Guazzaroni

**Affiliations:** ^1^Department of Biochemistry and Immunology, Faculdade de Medicina de Ribeirão Preto, Universidade de São Paulo, São Paulo, Brazil; ^2^Department of Chemistry, Faculdade de Filosofia, Ciências e Letras de Ribeirão Preto, Universidade de São Paulo, São Paulo, Brazil; ^3^Department of Cellular and Molecular Biology, Faculdade de Medicina de Ribeirão Preto, Universidade de São Paulo, São Paulo, Brazil; ^4^Department of Biology, Faculdade de Filosofia, Ciências e Letras de Ribeirão Preto, Universidade de São Paulo, São Paulo, Brazil

**Keywords:** GH3 β-glucosidase, functional metagenomics, 5-hydroxymethyl furfural, ethanol-stimulated enzyme, synergistic effect, bioethanol

## Abstract

Beta-glucosidases are key enzymes involved in lignocellulosic biomass degradation for bioethanol production, which complete the final step during cellulose hydrolysis by converting cellobiose into glucose. Currently, industry requires enzymes with improved catalytic performance or tolerance to process-specific parameters. In this sense, metagenomics has become a powerful tool for accessing and exploring the biochemical biodiversity present in different natural environments. Here, we report the identification of a novel β-glucosidase from metagenomic DNA isolated from soil samples enriched with decaying plant matter from a Secondary Atlantic Forest region. For this, we employed a functional screening approach using an optimized and synthetic broad host-range vector for library production. The novel β-glucosidase – named Lfa2 – displays three GH3-family conserved domains and conserved catalytic amino acids D283 and E487. The purified enzyme was most active in pH 5.5 and at 50°C, and showed hydrolytic activity toward several *p*NP synthetic substrates containing β-glucose, β-galactose, β-xylose, β-fucose, and α-arabinopyranose, as well as toward cellobiose. Lfa2 showed considerable glucose tolerance, exhibiting an IC_50_ of 300 mM glucose and 30% of remaining activity in 600 mM glucose. In addition, Lfa2 retained full or slightly enhanced activity in the presence of several metal ions. Further, β-glucosidase activity was increased by 1.7-fold in the presence of 10% (v/v) ethanol, a concentration that can be reached in conventional fermentation processes. Similarly, Lfa2 showed 1.7-fold enhanced activity at high concentrations of 5-hydroxymethyl furfural, one of the most important cellulase inhibitors in pretreated sugarcane bagasse hydrolysates. Moreover, the synergistic effect of Lfa2 on *Bacillus subtilis* GH5-CBM3 endoglucanase activity was demonstrated by the increased production of glucose (1.6-fold). Together, these results indicate that β-glucosidase Lfa2 is a promissory enzyme candidate for utilization in diverse industrial applications, such as cellulosic biomass degradation or flavor enhancement in winemaking and grape processing.

## Introduction

Conversion of lignocellulosic biomass into biofuels is a promising alternative to replace fossil fuels derived from non-renewable energy sources. Lignocellulose consists primarily of cellulose (40–60%), hemicelluloses (20–40%) and lignin (10–25%) (McKendry, 2002). Cellulose is a linear polymer of D-glucose subunits bound by β-1,4-glycosidic linkages with a polymerization degree ranging from few hundreds of D-glucose units to more than thousands. Hydroxyl groups of D-glucose units interact by hydrogen bonds resulting in a compact crystalline structure. As a result, although cellulose is the most recalcitrant material present in plant cell walls, it also has a substantial potential as a bioethanol production source (Sun et al., 2016).

The classical cellulose degradation framework for fungi includes: (i) the endo-1,4-β-glucanases (commonly found in GH families 5, 9, 44, and 45) which cleave the inner bonds of cellulose chain; (ii) exo-1,4-β-glucanases or cellobiohydrolases (most often from GH families 6, 7, 9, and 48) which release cellobiose molecules from either the reducing or non-reducing ends of a cellulose polymer, and (iii) the β-glucosidases (for example, belonging to the GH1 and GH3 families), which hydrolyze cellobiose in glucose molecules (Kubicek, 1992; Nidetzky et al., 1994; van den Brink and de Vries, 2011; Glass et al., 2013; Lombard et al., 2014). A limiting step in enzymatic cellulose saccharification is the conversion of oligosaccharides and cellobiose, which are products of endoglucanases and cellobiohydrolases hydrolysis, into glucose, and it has been demonstrated that the reaction products inhibit activities of most cellobiohydrolases and endoglucanases (Gruno et al., 2004; Xin et al., 2015; Yamamoto and Tamaru, 2016). In this context, β-glucosidases reduce cellobiose inhibition by hydrolyzing cellobiose into glucose, playing an important role in cellulose degradation process and acting as a key rate-limiting enzyme (Wojtusik et al., 2017). Further, synergism between cellulases plays an important role in biomass degradation and it has been reported for many cellulolytic enzyme systems, including free enzymes and also those that are part of cellulosomes (Beukes et al., 2008; Doi, 2008; Hu et al., 2013; Yang M. et al., 2016; Zhang et al., 2017). Quantitatively, when the combination of two enzymes is more efficient than the sum of each enzyme activities acting separately, the two enzymes show synergy. Due to the synergistic effect, one enzyme is able to accelerate the action of the other, with a consequent increase in hydrolysis yield (Saini et al., 2014).

Currently, one of the bottlenecks for second-generation bioethanol production is the high cost (Brijwani et al., 2010; Koppram et al., 2014) and the low efficiency of enzymes required for the hydrolysis of cellulosic materials into fermentable sugars (Li et al., 2016). It means that there is an increasing demand for new strategies to reduce process costs and for new biocatalysts with improved properties for industrial applications, such as high catalytic efficiency, increased stability at high temperatures and certain pHs, biocatalysts that are not inhibited by the product, as well as not inhibited by toxic compounds resulting from lignocelluloses pretreatments (Ximenes et al., 2010, 2011; Sun et al., 2016). According to Ramos et al. (2016), the second generation ethanol production expenses with enzymes can be about 15% of the total alcohol production cost. In this way, many efforts have been made in order to enhance the efficiency of enzyme production and the activity of these enzymes, as well as in finding new enzymes with such features (Ramos et al., 2016). In this context, metagenomics allows the identification of new enzymes with specific activities without the need of previous isolation and cultivation of microorganisms, which opens the door to the huge biochemical potential of most of the microbial life existing in environments of interest (Guazzaroni et al., 2015; Alves et al., 2017). Several examples reported in literature showed the identification of new cellulases and other enzymes from different environments through metagenomic approaches (Mori et al., 2014; Montella et al., 2015; Yang C. et al., 2016; Zhou et al., 2016; Lewin et al., 2017; Tiwari et al., 2017, 2018; Dadheech et al., 2018; Lee et al., 2018).

Enzymes presenting tolerance to organic solvents, such as ethanol, are preferred candidates for enzymatic hydrolysis in processes such as simultaneous saccharification and fermentation (SSF), used in the production of second generation bioethanol, in which ethanol concentrations may reach up to 8% (w/v) (Liu et al., 2016; Qin et al., 2018). In this context, ethanol-tolerant cellulases are interesting enzymes, once they could remain active in these conditions. Furthermore, enzymes that are tolerant to toxic compounds, such as 5-hydroxymethyl furfural (5-HMF), produced during biomass pretreatments, are preferably desired.

Here, we report the identification and biochemical characterization of a novel ethanol-and 5-HMF-stimulated β-glucosidase, using an activity-based metagenomic screening strategy. For this, we used DNA from a microbial community inhabitant of soil samples enriched with decaying plant matter from a Brazilian Secondary Atlantic Forest region. The rationale behind this choice was that environmental biomass-rich soil samples should also be enriched in genes encoding for cellulases. Additionally, a synthetic broad host-range vector was used for library generation and for subsequently confirming, in a straightforward manner, the enzyme activity in other hosts more suitable than *Escherichia coli* for industrial processes. In this sense, we showed Lfa activity against several substrates in *Pseudomonas putida*, a natural occurring soil bacterium with high metabolic versatility, robustness and significant potential for biotechnological applications (Wierckx et al., 2005; Craig et al., 2010; Beuttler et al., 2011; Van Duuren et al., 2011; Yu et al., 2016). Moreover, Lfa showed enhanced activity in harsh operating conditions, such as in presence of one of the most toxic inhibitory by-products from pretreated lignocellulose and also in 10% ethanol. Finally, we applied Lfa2 to the hydrolysis of a commercial polymeric substrate (carboxymethyl cellulose), in combination with GH5-CBM3 endoglucanase from *Bacillus subtilis*, to demonstrate its efficiency for enhancing the production of glucose.

## Materials and Methods

### Metagenomic Library Construction and β-Glucosidase Screening

The soil sample was obtained on July 2015 from a Secondary Atlantic Forest region at University of São Paulo (Ribeirão Preto, São Paulo, Brazil; 21°09′58.4″S, 47°51′20.1″W, at an altitude of 540 m) which was naturally enriched with decaying plant matter and was stored at -20°C until DNA extraction. For the library construction, metagenomic DNA was extracted using the UltraClean^TM^ Soil DNA isolation kit (Mo Bio, United States), partially digested using *Sau*3AI and fragments ranging from 2 to 7 kb were recovered directly from an agarose gel and ligated into a dephosphorylated and *Bam*HI-digested pSEVA232 vector (Silva-Rocha et al., 2013) (for vector map, see Supplementary Figure S4A). The resulting plasmids were transformed by electroporation in *E. coli* DH10B cells (for detailed workflow, see Supplementary Figure S3). The resulting metagenomic library (named LFA-USP3) was analyzed for the percentage of plasmids bearing metagenomic DNA and average insert size.

Screening for β-glucosidase activity was performed by plating LFA-USP3 metagenomic library into LB-agar plates supplemented with kanamycin (50 μg/ml) and streptomycin (50 μg/ml) and containing 0.15% (w/v) esculin hydrate and 0.03% (w/v) ferric chloride as described by Eberhart in 1964 (Eberhart et al., 1964). *E. coli* clones were incubated at 37°C for 24 h and further incubation at 30°C for 4 days was performed. Positive clones were identified by the appearance of dark halos surrounding the colonies. Plasmids of potential positive colonies were extracted from individual clones, retransformed into *E. coli* DH10B cells and plated on LB-agar plates containing 0.15% esculin hydrate and 0.03% ferric chloride to confirm the phenotype. Thus, one positive clone was confirmed, herein named pLFA2 (for vector map, see Supplementary Figure S4B), and its DNA insert was fully sequenced by using an Applied Biosystems 3500xL Genetic Analyzer sequencer (Applied Biosystems, United States).

### *In silico* Analysis and 3D-Structure Model Generation

The putative ORFs were predicted using ORF Finder program available in https://www.ncbi.nlm.nih.gov/orffinder/. ORF nucleotide sequences were translated to amino acid sequences using Translate tools program^1^ and putative amino acid sequences were analyzed using BlastP against non-redundant databases. Amino acid sequences were further analyzed for protein domains using Pfam database^2^, for signal peptide sequences using SignalP server^3^ and for physical and chemical parameters using ProtParam tool^4^. The 3D-structure was generated by SWISS-MODEL server using the automated mode^5^ (Schwede et al., 2003; Benkert et al., 2011; Waterhouse et al., 2018) and the catalytic site prediction in complex with glucose was generated by the ITASSER server^6^ (Zhang, 2008; Roy et al., 2010; Yang et al., 2014). All structures were analyzed using the PyMol software^7^. Multiple alignment of β-glucosidase with other GH3 family members was performed using Clustal Omega server^8^ and the multiple alignment printing was generated by the BoxShade server^9^. Twenty-seven amino acid sequences from characterized β-glucosidases were recovered from CAZy database^10^ to perform a phylogenetic reconstruction analysis along with the amino acid sequence of the Lfa2 using MEGA 6.0 software (Kumar et al., 1994; Tamura et al., 2013). Neighbor-joining statistical method was applied and accuracy of phylogenetic analysis was predicted by using 1,000 bootstrapping replications.

### Cloning, Gene Expression and Protein Purification

The putative β-glucosidase gene, named *lfa*2, was amplified by PCR using the primers: 5′ GACT**GGATCC**ATGAAATCCAGACTCGTAGCC 3′ containing a *Bam*HI restriction site (in bold) and 5′ AGTC**CTCGAG**TCACTTTGACGACGATAGCTC 3′ containing a *Xho*I restriction site (in bold) and using the pLFA2 plasmid as template. PCR conditions were as follows: initial denaturation at 98°C for 30 s, followed by 30 cycles of 98°C for 10 s, 65°C for 20 s, 72°C for 60 s and a final extension step of 72°C for 5 min. Then, the PCR product was digested with *Bam*HI and *Xho*I restriction enzymes and ligated into a pET28a vector previously digested by the same enzymes. The resulting clone was fully sequenced to check for the absence of mutations and transformed by electroporation into *E. coli* Rosetta (DE3) cells.

For expression and purification experiments, a single colony of *E. coli* Rosetta (DE3) cell harboring the resulting plasmid pET28a-lfa2 was grown in LB containing kanamycin (50 μg/ml) and chloramphenicol (36 μg/ml) at 37°C with shaking until to an *A*_600 nm_ of ∼0.8. Then, protein expression was induced by 0.1 mM IPTG followed by 18 h incubation at 18°C. Cells were harvest by centrifugation at 4,000 rpm for 30 min and cell pellet was resuspended in 6 mL of lysis buffer [30 mM HEPES pH 7.4, 300 mM NaCl, 1 mM PMSF, 1% (v/v) Triton-X and 20 mM imidazole]. After disruption by sonication, cell debris were collected by centrifugation at 10,000 rpm for 30 min and the supernatant was loaded on a nickel-charged HisTrap HP affinity column (GE Healthcare, United Kingdom). Protein purification was performed by using 30 mM HEPES buffer pH 7.4, 300 mM NaCl and increasing imidazole concentrations (40 and 80 mM). Finally, protein was eluted with the same buffer containing 300 mM imidazole. All fractions were collected and samples were analyzed by 12% SDS-PAGE (Laemmli, 1970). Purified protein concentration measurements were performed according to Read and collaborators using bovine serum albumin as standard (Read and Northcote, 1981).

### Biochemical Characterization

Biochemical properties of the β-glucosidase were determined using the purified enzyme and all experiments were performed in triplicate with suitable controls. Specific activity (U/mg) of β-glucosidase was determined at 50°C using 3 mM of pNP-based substrates including pNP-β-D-glucopyranoside (pNPβGlu), pNP-β-D-galactopyranoside (pNPβGal), pNP-β-D-xylopyranoside (pNPβXyl), pNP-α-D-xylopyranoside (pNPαXyl), pNP-β-D-fucopyranoside (pNPβFuc), pNP-β-D-mannopyranoside (pNPβMan), pNP-α-L-arabinopyranoside (pNPαAra) in 50 mM of citrate-phosphate buffer pH 5.5 and 0.3 μg of enzyme in a final volume of 30 μL. Reactions were stopped by addiction of 30 μL of a Na_2_[B_4_O_5_(OH)_4_] saturated solution and the amount of *p*NP released were measured in a 96-well plate at 405 nm in a xMark Microplate Spectrophotometer (Bio-Rad). One unit (U) was defined as the amount of β-glucosidase required to release 1 μmol of *p*NP per minute under assay conditions. For cellobiose, β-D-lactose, maltose and sucrose the amount of glucose released was measured using a glucose oxidase-based kit (Labtest, BR), following the manufacturer recommendations. Kinetic parameters (*K*_M_, *V*_max_, and *k*_cat_) were determined at 50°C in a substrate range of 0 to 15 mM for pNP-β-D-glucopyranoside and of 0 to 150 mM for cellobiose in 50 mM of citrate-phosphate buffer pH 5.5 and 0.5 μg of β-glucosidase. The data were analyzed with the Graph Prism 5.0 software (Graph-Pad Prism Software, United States).

Effect of temperature on enzyme activity was evaluated in temperatures ranging from 25 to 70°C in 50 mM of citric acid-phosphate buffer pH 5.5. For optimal pH determination, we used the range 2.0–8.0 at 50°C, using 50 mM of citric acid-phosphate buffer. Results were expressed in relative activity percentage according to either the optimum pH or optimum temperature.

The effects of several different compounds on β-glucosidase (0.2 μg) activity were analyzed using 4 mM of pNP-β-D-glucopyranoside as substrate. In these assays, the chelating agent ethylenediaminetetraacetic acid (EDTA) was tested at the final concentration of 10 mM. Each divalent metal ions Mg^2+^, Co^2+^, Mn^2+^, Cu^2+^, Ca^2+^, Ni^2+^, Zn^2+^, Fe^2+^ (using their respective salts: MgCl_2_, CoCl_2_, MnSO_4_, CuSO_4_, CaCl_2_, NiSO_4_, ZnSO_4_, and FeSO_4_) were tested at the final concentration of 10 mM, as well. Ethanol and DMSO were assayed each one at 10, 25, and 50% (v/v) and NaCl was added at final concentrations of 250 mM and 1 M. Ethanol assays were performed in tightly closed tubes, in order to avoid ethanol evaporation. All reactions were performed in 50 mM of citric acid-phosphate buffer pH 5.5 at 50°C in a tabletop heater. Control assays were performed using 4 mM of pNP-β-D-glucopyranoside in 50 mM of citric acid-phosphate buffer pH 5.5 at 50°C without any addictives.

Similarly, the inhibitory effect of different concentrations of vanillin (0–2%), 4-hydroxybenzoic acid (4-HBA) (0–2%), furfural (0–1%) and 5-hydroxymethyl furfural (5-HMF) (0–1%) and the inhibitory effect of different concentrations of glucose (0–1.35 M) were determined by incubating the purified β-glucosidase (0.2 μg) with 4 mM of pNP-β-D-glucopyranoside in 50 mM of citric acid-phosphate buffer pH 5.5 at 50°C. Likewise, control assays were performed using 4 mM of pNP-β-D-glucopyranoside in 50 mM of citric acid-phosphate buffer pH 5.5 at 50°C without any addictives. The IC50 is defined as the concentration of glucose that inhibited 50% of enzymatic activity when compared with the control (without glucose). All experiments were analyzed in percentage of relative activities according to the control assay (without addictives).

### Hydrolysis of Commercial Polymeric Substrate

Hydrolysis of carboxymethyl cellulose sodium salt (CMC) medium viscosity (Sigma, United States) by a GH5-CBM3 endoglucanase (*Bs*Cel5A) from *B. subtilis* 168 (in crude enzymatic extract) and β-glucosidase Lfa2 was performed in a 200 μL total volume containing 0.5% of CMC (w/v) in 50 mM of citric acid-phosphate buffer. Plasmid pSEVA242-*Bs*Cel5A was transferred to *E. coli* DH10B and a single colony was grown overnight in LB medium containing kanamycin (50 μg/ml). Overnight culture was used to inoculate 100 mL of LB medium and the culture was incubated at 37°C, 120 rpm for 24 h. Cells were collected by centrifugation and resuspended in 1 mL of 0.1 M citric acid-phosphate buffer pH 7.4 and disrupted by sonication. Cell debris were collected by centrifugation and 10 μL (10 U of *Bs*Cel5A endoglucanase per gram of CMC) of the crude protein extract was added to the reaction mixture which was incubated at 40°C per 45 min. Then, the reaction was supplemented with 20 μL (85 U of β-glucosidase per gram of CMC) of the purified β-glucosidase and incubated for further 1h. Glucose released was measured using a glucose oxidase-based kit (Labtest, BR). The synergism degree was defined as the ratio of the glucose amount released by the action of both enzymes to the sum of the glucose amount released by each enzyme independently.

### Evaluation of β-Glucosidase Activity in Crude Protein Extracts From Different Hosts

In order to evaluate β-glucosidase activity expressed in different hosts, pLFA2 plasmid was transferred to *P. putida* KT2440 and *E. coli* DH10B. Overnight cultures of each bacteria harboring pLFA2 plasmid were used to inoculate 100 mL of LB medium. Cultures were incubated at 30°C, 120 rpm for 48 h. Cultures of *P. putida* KT2440 and *E. coli* DH10B harboring pSEVA232 empty vector were used as negative controls (experiments with *p*NPβGlu, *p*NPβXyl, and *p*NPβFuc). When experiments were performed toward *p*NPβGal, cells of both bacteria without plasmid were used as negative controls, since pSEVA232 empty vector harbor the lacZα gene fragment, which encodes a β-galactosidase that could interfere in results interpretation. Cells were collected by centrifugation, resuspended in 1 mL of 0.1 M citric acid-phosphate buffer pH 7.4 and disrupted by sonication. Cell debris were collected by centrifugation and 5 μL of the crude protein extracts were used to compare specific activity of β-glucosidase expressed by two different hosts in 2 mM of different *p*NP-based substrates.

### Statistical Analysis

Tukey test at 5% significance level was accomplished by using the software Statistic 7.0.

## Results

### Generation and Evaluation of LFA-USP3 Metagenomic Library and β-Glucosidase Screening

**FIGURE 1:**
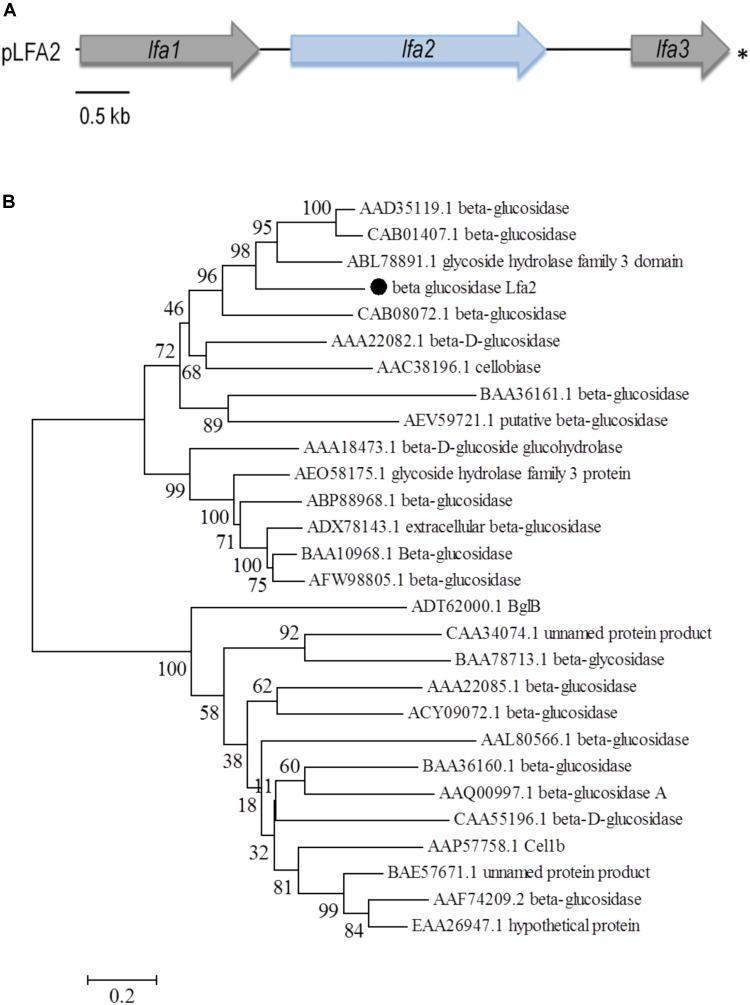
Schematic representation of the ORFs identified in the pLFA2 plasmid and phylogenetic analysis of β-glucosidase Lfa2. **(A)** The complete metagenomic insert is shown. Arrows display the location, length and transcriptional orientation of the ORFs. Lfa2 ORF is showed as a light blue arrow. Asterisk indicates incomplete ORFs. **(B)** Phylogenetic tree of 28 candidate sequences using the Neighbor-Joining (NJ) method. Amino acid sequences were recovered from GenBank as indicate by accession numbers as follows: AAD35119.1 (*Thermotoga maritima* MSB8), CAB01407.1 (*Thermotoga neapolitana*), ABL78891.1 (*Thermofilum pendens* Hrk 5), MH397474 (beta glucosidase Lfa2), CAB08072.1 (*Clostridium stercorarium*), AAA22082.1 (*Agrobacterium tumefaciens*), AAC38196.1 (*Cellulomonas biazotea*), BAA36161.1 (*Bacillus* sp.), AEV59721.1 (uncultured bacterium), AAA18473.1 (*Trichoderma reesei*), AEO58175.1 (*Thermothelomyces thermophila* ATCC 42464), ABP88968.1 (*Penicillium brasilianum*), ADX78143.1 (*Aspergillus fumigatus*), BAA10968.1 (*Aspergillus aculeatus*), AFW98805.1 (*Aspergillus niger*), ADT62000.1 (bacterium enrichment culture), CAA34074.1 (*Sulfolobus solfataricus*), BAA78713.1 (*Thermococcus kodakarensis* KOD1), AAA22085.1 (*Agrobacterium* sp.), ACY09072.1 (uncultured bacterium [2]), AAL80566.1 (*Pyrococcus furiosus* DSM 3638), BAA36160.1 (*Bacillus* sp.), AAQ00997.1 (*Clostridium cellulovorans*), CAA55196.1 (*Avena sativa*), AAP57758.1 (*Trichoderma reesei*), BAE57671.1 (*Aspergillus oryzae* RIB40), AAF74209.2 (*Aspergillus niger*), EAA26947.1 (*Neurospora crassa*). Numbers on nodes correspond to percentage bootstrap values for 1,000 replicates.

A metagenomic library, herein named LFA-USP3, was generated from an environmental soil sample collected at South America, from a Secondary Atlantic Forest at the University of São Paulo (Ribeirão Preto, São Paulo, Brazil) enriched in decaying leaves from *Anadenanthera* sp. Metagenomic DNA was partially digested and cloned into the pSEVA232 vector (Silva-Rocha et al., 2013). The library comprised around 257 Mb of environmental DNA distributed into approximately 63,000 clones (100,000 clones – total library – which 63% carrying inserts) carrying insert fragments with an average size of 4.1 kb, thus presenting a number of genomes estimated in about 57; assuming 4.5 Mb per genome (Raes et al., 2007). In total, approximately 216,000 clones from LFA-USP3 library were screened for β-glucosidase activity using an esculin-based plate assay. We identified some dark halo-forming colonies which were selected for DNA plasmid extraction and retransformation into *E. coli* DH10B cells for verifying phenotype maintenance. After retransformation, one colony recovered the phenotype observed in the initial screening. This colony was selected for DNA plasmid extraction and its metagenomic insert was fully sequenced using a primer walking approach. The insert sequence of approximately 5.8 kb presented three open reading frames, designated as Lfa1, encoding for a protein displaying a conserved domain for the AXE1 superfamily (Acetyl Xylan Esterase superfamily), Lfa2, which encodes a potential GH3 glycosyl hydrolase, and Lfa3, encoding a truncated carboxypeptidase (Figure 1A and Table 1). Lfa2 consists of a 2,358-bp fragment encoding 785 amino acids. It shares the highest amino acid sequence identity (59%) with a non-characterized GH3 glycosyl hydrolase from *Pyrinomonas methylaliphatogeness* (Genbank No. WP_041974888.1), a thermophilic and acidophilic Gram-negative bacteria from soil. The new sequence is available at Genbank with accession number MH397474.

**Table 1:** Description of the ORFs contained in pLFA2 plasmid and their sequence similarities.

Plasmid name [insert (bp)]	% G + C	ORF^a^	Length (aa)^b^	Closest similar protein (length in aa)	Organism	*E*-value	Identity (%)	Putative function	No. Of TMH^c^	Signal Peptide
pLFA2 (5,868)	53,2	1	577	hypothetical protein A2W03_04630 [OGD23027.1] (641)	Candidatus *Aminicenantes* bacterium RBG_16_63_16	<5.00E-133	56	Acetyl xylan esterase	0	No
		2	785	Glycosyl hydrolase family 3 [WP_041974888.1] (787)	*Pyrinomonas methylaliphatogenes*	<5.00E-133	59	Glycosyl hydrolase family 3	0	Yes^d^
		3^∗^	385^∗∗^	Carboxypeptidase [OGU07865.1] (461)	*Gemmatimonadetes* bacterium RBG 16668	5.00E-133	62	Carboxypeptidase	1	No


**^*a*^Open reading frame. ^*b*^Amino acids. ^*c*^Number of transmembrane helices (TMH) predicted by using the TMHMM program (http://www.cbs.dtu.dk/services/TMHMM-2.0/). ^*d*^According to SignalP software (http://www.cbs.dtu.dk/services/SignalP/), the clivage site is between aminoacids 22 and 23; considering an analysis for Gram-negative bacteria. ^∗^Asterisks indicate incomplete ORFs and ^∗∗^proteins truncated at C-terminal end*.*

Phylogenetic analysis of β-glucosidase Lfa2 (Figure 1B) with other 27 β-glucosidase amino acid sequences showed the presence of three different groups in the phylogenetic tree: (i) GH3 β-glucosidases from bacteria and archaea, (ii) GH3 β-glucosidases from fungi and (iii) GH1 β-glucosidases from bacteria, archaea and fungi. In accordance with previous bioinformatic analyses, Lfa2 is located in the first group, indicating that it is possibly sharing some functional and structural features with proteins positioned in this group.

### Sequence and 3D-Structure Analysis of the β-Glucosidase Lfa2

**FIGURE 2:**
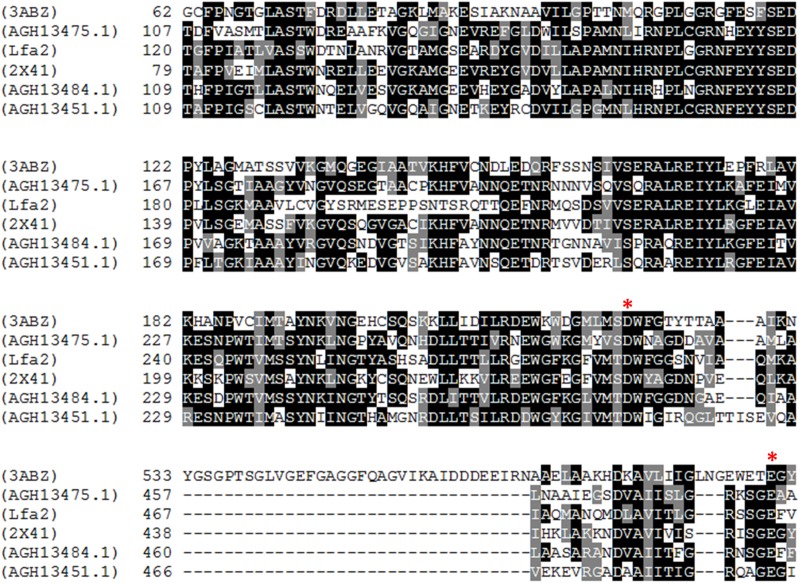
Multiple alignment of sequences from β-glucosidase Lfa2 and other GH3 family β-glucosidases. Amino acid sequence alignment of the metagenomic β-glucosidase Lfa2 was carried out using the Clustal Omega server. Results were shaded using BOXSHADE (black background = strictly conserved; gray or white background = conservatively substituted or non-conserved). Asterisks indicate the nucleophile residue D283 and the acid/base amino acid residue E487. Full species names and Genbank, PDB or NCBI IDs of the β-glucosidases sequences are shown as follow: *Kluyveromyces marxianus* PDB ID: 3ABZ (3ABZ); metagenomic β-glucosidases from cow rumen NCBI accession numbers: JX163905, JX163906, and JX163904 (AGH13475.1, AGH13484.1, and AGH13451.1); *Thermotoga neapolitana* PDB ID: 2X41 (2X41) and metagenomic β-glucosidaseLfa2 from Secondary Atlantic Forest soil (this study) Genbank accession number: MH397474 (Lfa2).

Multiple amino acid sequence alignment of Lfa2 with other members of the GH3 family is shown in Figure 2. All proteins used in the alignment have shown activity against cellobiose in previous works and shared highly conserved sequences (Pozzo et al., 2010; Yoshida et al., 2010; Pozo et al., 2012; McAndrew et al., 2013). From this alignment, we found that the catalytic nucleophile (D283) and the acid/base amino acid (E487) residues were conserved (see Supplementary Material for full amino acid alignment, Supplementary Figure S1), which are also fully conserved in members of the GH3 family (Dan et al., 2000; Pozzo et al., 2010; Yoshida et al., 2010; McAndrew et al., 2013; Thongpoo et al., 2013), consistent with their key roles as catalytic amino acids.

**FIGURE 3:**
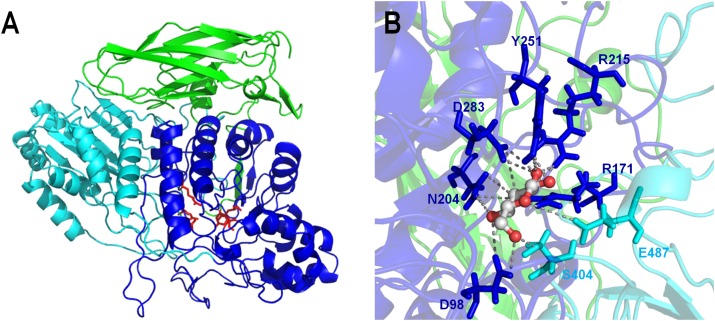
3D-structure model of the β-glucosidase Lfa2 characterized in this work. **(A)** Overall 3D-structure representation showing the three domains of the β-glucosidase: TIM barrel-like domain (in blue), α/β sandwich domain (in cyan) and the C-terminal fibronectin type III domain (in green). Side chains of the catalytic amino acids and amino acids involved in substrate binding are shown in red. **(B)** Catalytic site representation in complex with glucose. Amino acid side chains are represented by sticks, indicating the conserved catalytic amino acids D283 and E487 and other amino acids involved in substrate recognition and stabilization (D98, R171, N204, R215, Y251, and S404).

The predicted 3D-structure model of the Lfa2 (89% of coverage, residues 34–762) was obtained from Swiss Model server based on the crystal structure of the β-glucosidase 3B from *Thermotoga neapolitana* (PDB: 2x42), which shares 49.2% of amino acid identity and 44% of amino acid similarity with Lfa2. The resulting GMQE and QMEAN scores were 0.7 and -1.38, respectively (Benkert et al., 2011; Waterhouse et al., 2018). As shown in Figure 3A, the predicted 3D-structure is composed by an α/β triose phosphate isomerase (TIM) barrel-like domain, an α/β sandwich domain and a C-terminal fibronectin type III domain, according to other β-glucosidases from GH3 family (Pozzo et al., 2010; Suzuki et al., 2013). Side chains of the important functional amino acids involved in substrate recognition and hydrolysis D98, R171, N204, R215, Y251, and S404 are highlighted in red. The TIM barrel-like domain and the α/β sandwich domains play an important role in the active-site organization; the last domain may be involved with substrate interaction and could affect enzyme stability (Pozzo et al., 2010). Additionally, the interaction analysis between a molecule of glucose in complex with Lfa2 showed that D283 (located in the TIM barrel-like domain) and E487 (positioned in the α/β sandwich domain) were conserved (Figure 3B).

### Biochemical Characteristics of the β-Glucosidase Lfa2

Lfa2 was overexpressed and purified from cell-free enzymatic extract by Ni^2+^ affinity chromatography. Molecular mass of the enzyme was estimated to be 84 kDa using the ProtParam server^11^, with a theoretical pI of 9.24. The purified recombinant protein appeared on SDS-PAGE electrophoresis gel as a band (see Supplementary Figure S2) between 66.2 and 116 kDa bands. Biochemical characterization of the Lfa2 was investigated using the purified recombinant enzyme.

**FIGURE 4:**
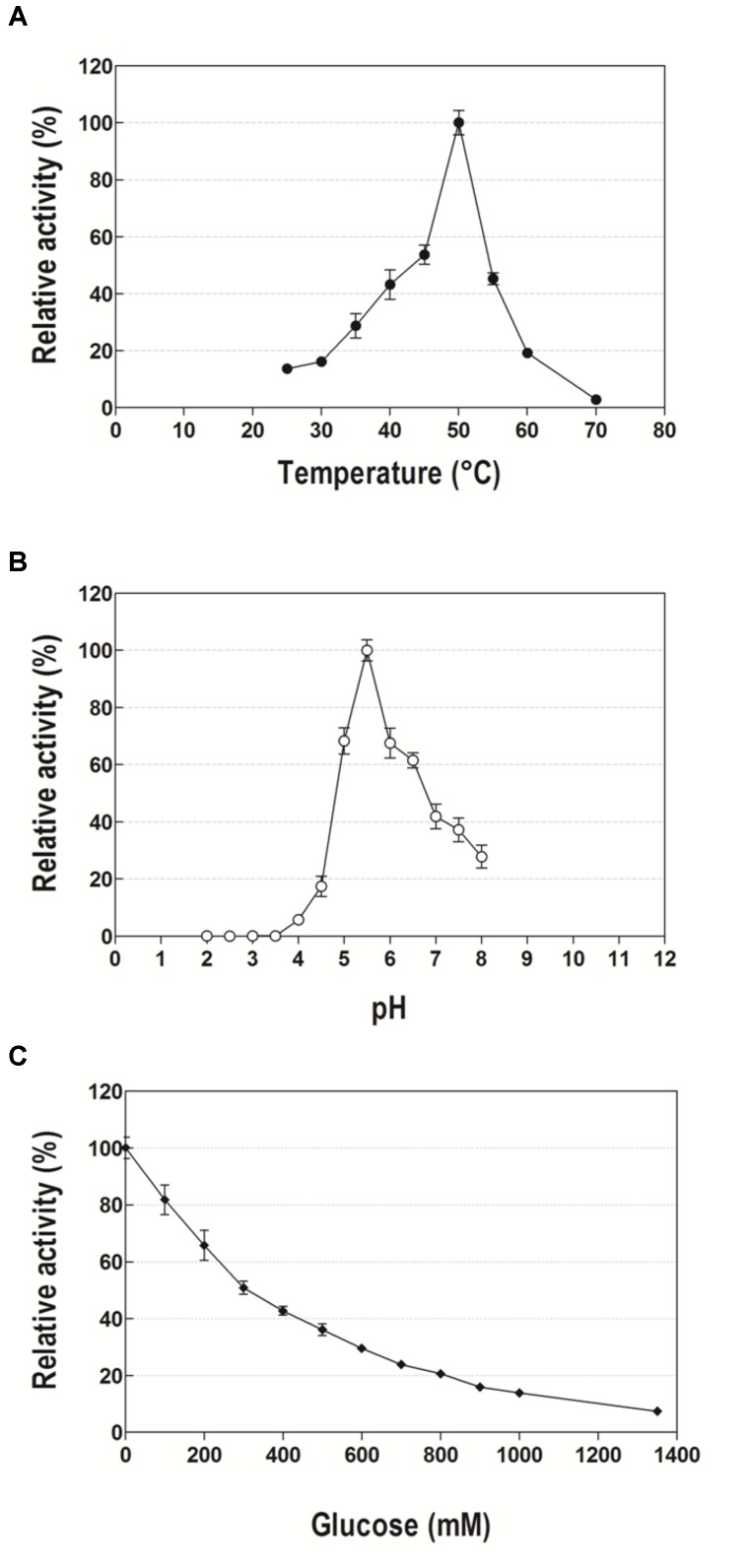
Effects of temperature, pH and glucose concentration on the activity of the β-glucosidase Lfa2. **(A)** Effect of temperature on the activity of Lfa2 was determined in pH 5.5 (citric acid-phosphate buffer 50 mM) in a range of temperature from 25 to 70°C. **(B)** Effect of pH on the activity of Lfa2 was determined in the range of pH 2.0–8.0 at 50°C using citric acid-phosphate buffer (50 mM). **(C)** Influence of increasing glucose concentrations (0–1.35 M) on Lfa2 activity was performed in citric acid-phosphate buffer 50 mM pH 5.5 at 50°C. All enzymatic properties were determined using pNPβG as substrate. All measurements were done in triplicates. Error bars show SD. Activity at 100% refers to a specific activity of 9.6 μmol min^-1^ mg^-1^.

The optimal temperature for Lfa2 was determined to be 50°C, while the enzyme activity was approximately 50% of the maximum activity at a range from 40 to 55°C, maintaining more than 20% of the maximum activity at a range from 35 to 60°C (Figure 4A). Lfa2 was most active at pH 5.5, retaining more than 60% of the maximum activity in a pH range from 5 to 6.5 (Figure 4B).

### Effect of Glucose on the Enzymatic Activity of Lfa2

In order to evaluate the effect of increasing glucose concentrations on Lfa2 activity, catalytic activity was measured using *p*NP-Glu as a substrate in presence of 0–1.35 M of glucose. As shown in Figure 4C, the enzyme presented considerable tolerance to glucose inhibition, presenting an IC_50_ of 300 mM glucose. When glucose concentrations were further increased, the enzyme activity was gradually inhibited, with 20% of remaining activity at higher glucose concentrations as 800 mM.

### Effects of Metal Ions, Organic Solvents, EDTA and Salt on Lfa2 Activity

The effect of divalent metal ions, organic solvents, chelating agent and salt on the enzymatic activity of Lfa2 were analyzed (Table 2). The enzymatic activity of Lfa2 was slightly increased in the presence of Mg^2+^, Co^2+^, and Mn^2+^ and strongly inhibited in the presence of Cu^2+^ and Fe^2+^. The relative enzymatic activity improved to 130.8 ± 4.3% (Mg^2+^) 134.1 ± 11.2% (Co^2+^) and 109.4 ± 0.3% (Mn^2+^), but it was reduced to 5.8 ± 1.3 and 17.4 ± 4.1% in presence of Cu^2+^ and Fe^2+^, respectively. The effects of Ca^2+^, Ni^2+^, and Zn^2+^ on the enzymatic activity were not significant. Chelating agent EDTA (ethylenediaminetetraacetic acid) reduced the relative activity to 79.3 ± 1.6%. On the other hand, relative activity was increased approximately 30% in presence of NaCl. Enzymatic activity was inhibited in presence of increasing concentrations of the organic solvent DMSO (dimethyl sulfoxide). Instead, in presence of 10% ethanol the relative enzymatic activity was improved in 70% relative to the control (without additives). Additionally, increasing concentrations of ethanol (25 and 50%) reduced enzyme activity to approximately 59 and 35%, respectively.

**Table 2:** Influence of metal ions, chelating agent, organic solvents and salt on Lfa2 activity.

Compound	Relative activity (%)
Control (no addictives)	100 ± 6.1
10 mM MgCl_2_	130.8 ± 4.3
10 mM CoCl_2_	134.1 ± 11.2
10 mM MnSO_4_	109.4 ± 0.3*
10 mM CuSO_4_	5.8 ± 1.3
10 mM CaCl_2_	105.7 ± 5.5*
10 mM NiSO_4_	92.1 ± 3*
10 mM ZnSO_4_	94.6 ± 8.8*
10 mM FeSO_4_	17.4 ± 4.1
10 mM EDTA	79.3 ± 1.6
250 mM NaCl	137.4 ± 6.5
1 M NaCl	135.9 ± 6.1
10% DMSO	86.6 ± 3.1*
25% DMSO	65.5 ± 3
50% DMSO	21.3 ± 1.2
10% EtOH	170.1 ± 1.7
25% EtOH	59.1 ± 2.4
50% EtOH	35.6 ± 0.8


**^∗^No significant difference when compared with the control (no addictives) as revealed by Tukey test, p < 0.05. Activity at 100% refers to a specific activity of 7.9 μmol min^-*1*^ mg^-*1*^ using pNPβGlu as substrate*.*

### Substrate Specificity and Kinetic Parameters of Lfa2

Under optimal pH and temperature parameters, the hydrolytic activity of Lfa2 toward various substrates was measured and the substrate specificity was determined (Table 3). Among the chromogenic substrates, β-glucosidase Lfa2 showed activity toward pNPβGlu, pNPβXyl, pNPαAra, pNPβFuc, and pNPβGal, while no activity was observed upon pNPαXyl and pNPβMan. Lfa2 showed activity toward cellobiose, but no detectable activity was observed toward other oligosaccharides tested. The enzyme showed to be more active toward pNPβGlu, followed by pNPβXyl and cellobiose which were approximately 25 and 10% of the maximum activity observed toward pNPβGlu. These results indicate that Lfa2 is a β-glucosidase with a broad specificity substrate range.

**Table 3:** Substrate specificity of Lfa2 for chromogenic substrates and oligossacarides.

Substrate (3 mM)	Specific activity (U/mg)
**Chromogenic substrates**	
pNPβGlu	8.09 ± 0.01^a^
pNPαAra	0.63 ± 0.09^cd^
pNPβXyl	2.1 ± 0.2^b^
pNPβGal	0.26 ± 0.04^e^
pNPβFuc	0.38 ± 0.01^de^
pNPαXyl	n.d^∗^
pNPβMan	n.d^∗^
**Oligosaccharides**	
Cellobiose	0.73 ± 0.03^c^
Maltose	n.d^∗^
Lactose	n.d^∗^
Sucrose	n.d^∗^


**^∗^n.d – activity not detected. ^*a,b,c,d,e*^Indicate significant difference between different specific activities for each substrate, as revealed by Tukey test, p < 0.05*.*

Enzymatic reaction rate regarding substrate concentration of pNPβGlu and cellobiose were tested and it followed the Michaelis–Menten kinetics. Then, half-saturation coefficient (*K*_M_), catalytic rate constant (*k*_cat_) and catalytic efficiency (*k*_cat_/*K*_M_) values were determined (Table 4). According to *k*_cat_/*K*_M_ values, Lfa2 showed greater efficiency for pNPβGlu (17.4 × 10^3^ s^-1^ M^-1^) when compared with cellobiose (3.02 × 10^2^ s^-1^ M^-1^).

**Table 4:** Kinetic parameters of the purified β-glucosidase Lfa2.

Substrate	*V*_máx_ (μmol min^-1^ mg^-1^)	*K*_M_ (mM)	*k*_cat_ (s^-1^)	*k*_cat_/*K*_M_ (s^-1^ M^-1^)
pNPβGlu	9.4 ± 0.2	0.76 ± 0.06	13.2 ± 0.2	17.4 × 10^3^
Cellobiose	16.2 ± 1.2	75.3 ± 11.6	22.8 ± 1.7	3.02 × 10^2^


### Effect of Lignocellulose-Derived Compounds on Lfa2 Activity

**FIGURE 5:**
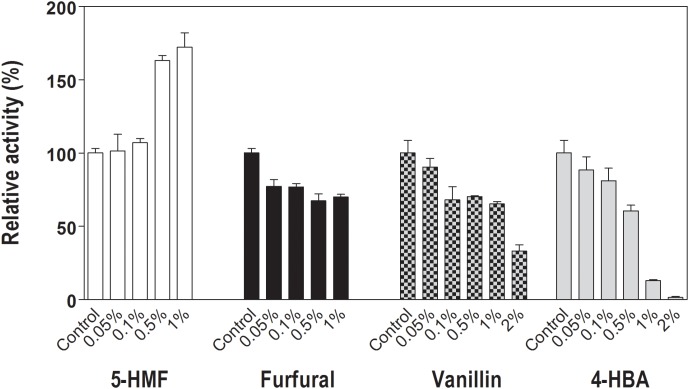
Relative activity of Lfa2 in different concentrations of lignocellulose pretreatment-derived compounds. Different concentrations of furfural (0–1%), 5-hydroxymethyl furfural (5-HMF) (0–1%), vanillin (0–2%), and 4-hydroxybenzoic acid (4-HBA) (0–2%) on Lfa2 activity were analyzed using 4 mM *p*NPβGlu as substrate in citric acid-phosphate buffer 50 mM pH 5.5 at 50°C. Control experiments were performed in absence of lignocellulose pretreatment-derived compounds. All measurements were done in triplicates. Error bars show SD. Activity at 100% refers to a specific activity of 7.9 μmol min^-1^ mg^-1^.

Effects of four potential cellulase inhibitors, furfural, 5-hydroxymethyl furfural (5-HMF), vanillin and 4-hydroxybenzoic acid (4-HBA) on Lfa2 activity were analyzed using 4 mM *p*NPβGlu as substrate. Different concentrations of furfural (0–1%), 5-HMF (0–1%), vanillin (0–2%), and 4-HBA (0–2%) showed contrasting effects on Lfa2 activity (Figure 5). For instance, Lfa2 activity was strongly inhibited in presence of 0.5 and 1% 4-HBA (enzymatic activity decreased approximately 40 and 90%, respectively), while 2% 4-HBA produced practically 100% inhibition. Additionally, 0.1% vanillin resulted in 30% of Lfa2 inhibition, whereas in the presence of 2% vanillin enzyme activity showed 70% of Lfa2 inhibition. Furfural displayed a slight effect on Lfa2 activity, inhibiting enzymatic activity no more than 30%, even in higher concentrations, as 1%. On the other hand, 5-HMF did not affect enzymatic activity in lower concentrations as 0.05 and 0.1%, whereas in higher concentrations, such as 0.5 and 1%, unexpectedly, 5-HMF activated Lfa2 activity, increasing *p*NPβGlu hydrolysis in 60 and 70%, respectively.

### Synergistic Effect of β-Glycosidase Lfa2 on *Bacillus subtili*s GH5-CBM3 Endoglucanase Activity

**FIGURE 6:**
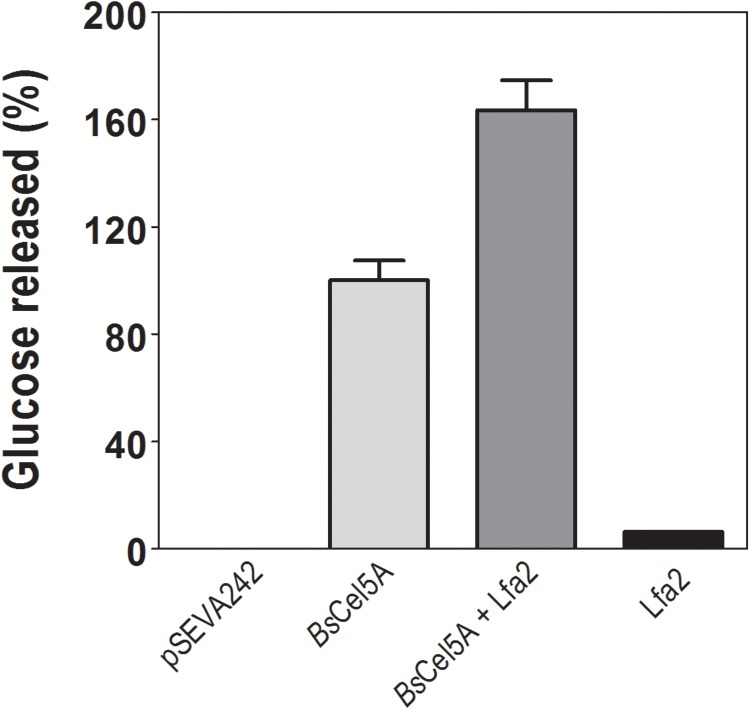
Synergistic effect of Lfa2 on endoglucanase GH5-CBM3 (Cel5A). Synergism was determined using carboxymethyl cellulose (CMC) as substrate. Plasmid pSEVA242 was used as the negative control of crude enzyme extract; BsCel5A, endoglucanase GH5-CBM3 (10 U per mg CMC); BsCel5A + Lfa2, endoglucanase GH5-CBM3 (at 10 U per mg CMC) supplemented with Lfa2 (at 85 U per mg CMC); Lfa2, 80 U per mg of CMC. All measurements were done in triplicates. Error bars show SD. Glucose released at 100% refers to 4.3 nmol of glucose released.

The synergistic effect of purified β-glycosidase Lfa2 and GH5-CBM3 (BsCel5A) endoglucanase of *B. subtilis* (in crude extract) was investigated using CMC as substrate. For this, enzymatic crude extract containing *Bs*Cel5A endoglucanase expressed from pSEVA242-*Bs*Cel5 plasmid and the purified Lfa2 were used. As shown in Figure 6, Lfa2 and crude enzymatic extract from empty plasmid pSEVA242 were unable to produce glucose from CMC. On the other hand, when combining endoglucanase activity of *Bs*Cel5A with β-glucosidase activity of Lfa2, the amount of glucose released was about 1.6-fold higher compared with endoglucanase alone. This result indicates that Lfa2 has positive effects on cellulose hydrolysis, acting in synergy with *Bs*Cel5A. The degree of synergism was estimated in 1.53-fold which was calculated according to the methodology in Section “Hydrolysis of Commercial Polymeric Substrate.”

### Evaluation of Lfa2 Activity in Crude Protein Extract Generated From Different Hosts

**FIGURE 7:**
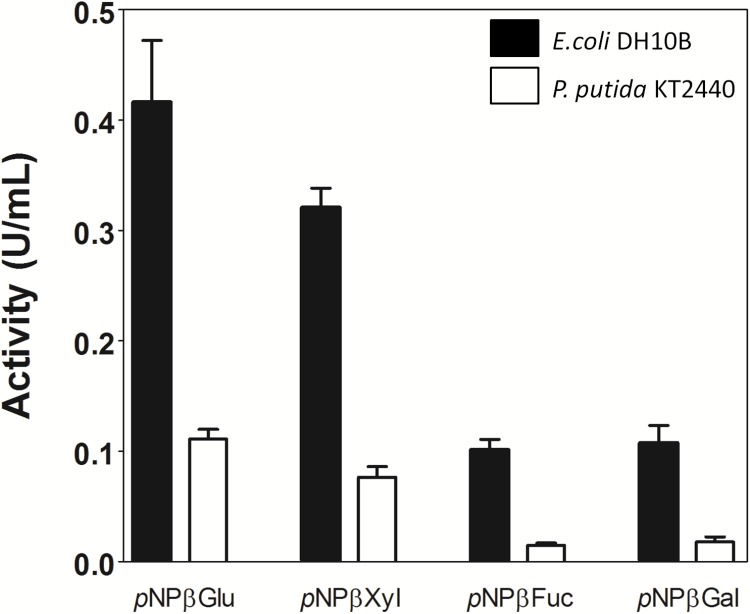
Activity of β-glycosidase Lfa2 from crude enzymatic extract of *Escherichia coli* DH10B and *Pseudomonas putida* KT2440 bearing plasmid pLFA2. Differences in specific activity of β-glucosidase Lfa2 from crude enzymatic extract produced in *E. coli* DH10B and *P. putida* KT2440 toward different *p*NP-derived substrates was determined using 2 mM of *p*NP-derived substrates in citric acid-phosphate buffer 50 mM pH 5.5 at 50°C. All measurements were done in triplicate. Error bars show SD.

As previously mentioned, a synthetic broad host-range vector was used for library production. Plasmid pSEVA232 has an origin of replication that can be recognized by a large number of Gram-negative hosts (Silva-Rocha et al., 2013). Thus, activity of Lfa2 was verified in crude extracts from *E. coli* DH10B and *P. putida* KT2440 harboring pLFA2 plasmid. As shown in Figure 7, different specific activities (U/mL) toward *p*NP-derived substrates were observed among both bacteria, being lower in crude extract from *P. putida* when compared with *E. coli*, probably due to differences in the protein expression levels in both hosts (Troeschel et al., 2012).

## Discussion

Metagenomic approaches have been successfully used in biotechnological fields, mining new biocatalysts for industrial purposes. In particular, several efforts have been made to find cellulases with relevant characteristics for specific parameters used in ethanol industry (Rees et al., 2003; Ferrer et al., 2005; Schröder et al., 2014; Garg et al., 2016; Lewin et al., 2017; Dadheech et al., 2018; Duque et al., 2018; Lee et al., 2018; Tiwari et al., 2018). Here, we report the identification and biochemical characterization of a novel ethanol- and 5-HMF-stimulated β-glucosidase, using a functional-based metagenomic screening strategy. For metagenomic library generation, the pSEVA232 synthetic broad host-range vector was used (Silva-Rocha et al., 2013). Thus, library construction in a wide-ranging host vector allowed us to confirm in a straightforward manner the enzyme activity in other hosts more suitable than *E. coli* for industrial applications. In this sense, we tested Lfa2 activity in *P. putida*, a metabolically robust bacterium with high reducing power output and low cellular maintenance demand (Wierckx et al., 2005; Craig et al., 2010; Beuttler et al., 2011; Van Duuren et al., 2011; Ng et al., 2015; Yu et al., 2016). We observed that Lfa2 was active against several substrates in both bacteria (Figure 7), but displayed different specific activities, probably due to particular expression levels of heterologous proteins in different hosts. Differences in plasmid copy number, codon usage, promoter and ribosome binding site recognition may lead to different expression levels in distinct hosts and it is difficult to predict which host strain may be the best choice for an heterologous protein expression (Terpe, 2006). In a recent study, expression of two reporter proteins was compared when expressed in *E. coli* BL21 and *P. putida* KT2440. Using a broad-host range shuttle vector based on PBBR1 origin of replication (same origin that plasmid pSEV232 used in this study), authors showed that plasmid copy number was similar in *E. coli* and *P. putida*, but expression of the reporter proteins in *P. putida* resulted in decreased enzymatic activities when compared with *E. coli* (Troeschel et al., 2012). Thus, a metagenomic library constructed in a vector that allows straightforward plasmid transfer to other bacterial hosts should simplify the process of identifying a proper host for ideal heterologous protein expression.

β-glucosidases are key enzymes involved in lignocellulosic biomass degradation for bioethanol production (Koppram et al., 2014; Liu and Cotta, 2015; Treebupachatsakul et al., 2016) which complete the final step during cellulose hydrolysis by converting cellobiose into glucose (Bhatia et al., 2002), a key rate-limiting step in cellulose degradation. Classification of glycosyl hydrolases based on amino-acid sequence similarities was proposed some years ago (Henrissat et al., 1991; Henrissat and Bairoch, 1996), and β-glucosidases were divided among two different families: GH1 and GH3. The amino acid sequence of Lfa2 displayed the highest sequence identity (59%) with a GH3 glycosyl hydrolase from *P. methylaliphatogeness* (Genbank No. WP_041974888.1). Furthermore, phylogenetic analysis showed that Lfa2 has a close relationship with GH3 β-glucosidases from bacteria and archaea. Thus, *in silico* and experimental results indicated that Lfa2 is a β-glucosidase that belongs to the GH3 family, presenting conserved domains and conserved catalytic amino acids of this family. The 3D-structure model revealed three conserved domains: α/β triose phosphate isomerase (TIM) barrel-like domain, an α/β sandwich domain and a C-terminal fibronectin-like type III domain, also present in other β-glucosidases from GH3 family (Pozzo et al., 2010; McAndrew et al., 2013; Suzuki et al., 2013). The two former domains play an important role in the active site formation (which is positioned at the interface of this two domains), since the catalytic residues (the nucleophile residue D283 and the acid/base amino acid residue E487) as well as other amino acids involved in substrate recognition, binding and/or stabilization (D98, R171, N204, R215, Y251, and S404) are conserved. The fibronectin-like type III domain possibly does not play a role in small substrates recognition, due to its position, but it could be involved in thermostability and binding in long polymeric substrates.

Regarding substrate specificity, β-Glucosidases can be divided into three different groups, namely, (1) aryl-β-glucosidases, which display strong affinity to aryl-β-glucosides; (2) true cellobiases, which hydrolyze only oligosaccharides and (3) broad substrate-range, that show activity on a large range of substrates (Rojas et al., 1995; Riou et al., 1998). The β-glucosidase identified in this study, which showed activity on a wide range of pNP substrates, such as pNPβGlu, pNPβXyl, pNPαAra, pNPβFuc, pNPβGal, and cellobiose, but presented no activity upon pNPαXyl, pNPβMan, sucrose, maltose, and lactose, belongs to the first group. Kinetic parameters of Lfa2 were measured and the enzyme exhibited catalytic efficiency coefficients (*k*_cat_/*K*_M_) of 17.4 × 10^3^ s^-1^ M^-1^ for pNPβGlu and 3.02 × 10^2^ s^-1^ M^-1^ for cellobiose. This values of catalytic efficiency are between some values of catalytic efficiency already reported for β-glucosidases from bacteria, fungi and metagenomes (we found values ranging from 2.9 × 10^4^ s^-1^ M^-1^ to 1.6 s^-1^ M^-1^ for *p*npβGlu) (Zanoelo et al., 2004; Karnchanatat et al., 2007; Joo et al., 2009; Yapi Assoi Yapi et al., 2009; Beloqui et al., 2010; Cobucci-Ponzano et al., 2010; Jiang et al., 2010, 2011; Naz et al., 2010; Fan et al., 2011; Kim et al., 2011, 2006; Bao et al., 2012; Haq et al., 2012; Zhao et al., 2012) (see Supplementary Table S2). On the other hand, these values are lower than catalytic efficiencies determined to the mainly commercial β-glucosidases (Seidle et al., 2004; Chauve et al., 2010). Additionally, Lfa2 showed considerable glucose tolerance capacity with an IC_50_ of 300 mM. Most microbial β-glycosidases are inhibited by glucose, which is an important limitation for its use in industry (Leite et al., 2008). High glucose concentrations may interfere directly or indirectly with substrate binding at the active site of the enzyme, reducing reaction rates (Singhania et al., 2013). It is worth mentioning that some glucose tolerant β-glucosidases were already isolated and characterized (Zanoelo et al., 2004; Pei et al., 2012; Zhao et al., 2013; Matsuzawa and Yaoi, 2017), presenting a value of IC_50_ higher than found for Lfa2 (Pei et al., 2012; Lu et al., 2013). For instance, Matsuzawa and Yaoi (2017) described a β-glucosidase stimulated by saccharides that presented more than sixfold of increased activity in presence of 250 mM glucose. Pei and collaborators, showed a β-glucosidase with an IC_50_ of 600 mM glucose. In other study, Lu and collaborators showed a β-glucosidase-tolerant enzyme with an IC_50_ of 1500 mM glucose. Many studies have suggested that glucose tolerance and stimulation of β–glucosidases properties are associated with transglycosylation activity (Uchiyama et al., 2013, 2015; Yang et al., 2015), so enzymes with these features could overcome the product inhibition by transglycosylation activity during the cellulose hydrolysis (Wang et al., 2017).

Notably, effect of ethanol on enzymatic activity is essential for β-glycosidase characterizations, since these enzymes are exposed to substantial concentrations of ethanol in a number of applications, such as the simultaneous saccharification and fermentation process (Garcia et al., 2015; Liu et al., 2016; Sun et al., 2016). Ethanol concentrations of 10% increased the enzymatic activity toward pNPβGlu by 1.7-fold compared with the activity without ethanol addition (Table 2). Moreover, increased ethanol concentrations were not able to completely inactivate Lfa2 activity, which remained with almost 60 and 35% of activity under 25 and 50% ethanol, respectively. It is worth mentioning that these activities are still high in comparison to other enzymes described as tolerant and/or activated by ethanol (Gueguen et al., 1996; Manzanares Rojas et al., 2000; Gosset and Martinez, 2012; Karnaouri et al., 2013; Garcia et al., 2015). Most β-glucosidases are inhibited in presence of ethanol, even in lower concentrations (Zhou et al., 2016; Matsuzawa and Yaoi, 2017). Though, some β-glucosidases activated by ethanol and/or ethanol-tolerant have been described (Parry et al., 2001; Muñoz-Gutiérrez et al., 2012; Karnaouri et al., 2013; Fang et al., 2016; Wu et al., 2018; Xue et al., 2018) (as showed in Supplementary Table S1). Uchiyama et al. (2013) described a metagenomic β-glucosidase activated 1.16-fold by ethanol 10% whereas 25% ethanol dramatically reduced enzyme activity. Parry et al. (2001) described a thermostable β-glucosidase from *Thermoascus aurantiacus* which was activated by 40% at 30% ethanol and inactivated at concentrations higher than 50% ethanol. A GH3 β-glucosidase with remarkable stimulation and tolerance to ethanol was characterized from *Myceliophthora thermophila* by Karnaouri et al. (2013). Enzyme activity was increased twofold in a range from 10 to 30% of ethanol. Considering that the final concentration of ethanol in a conventional fermentation process is approximately 10–15% (Koppram et al., 2014), enzymes with tolerance toward ethanol may be useful for application in saccharification processes for bioethanol production (Baffi et al., 2013).

Another interesting feature of Lfa2 is its behavior in presence of lignocellulose pretreatment-derived compounds. Some conditions used during lignocellulose pretreatment may lead to the generation of certain compounds that could affect the subsequent enzymatic hydrolysis (inhibiting enzymes) and fermentation (inhibiting microorganisms growth and survival) (Lavarack et al., 2002; Ximenes et al., 2011; Quéméneur et al., 2012). Generation of these compounds depends on the lignocellulose source, the type of pretreatment used and the conditions employed in pretreatment (Du et al., 2010; van der Pol et al., 2014). The main compounds generated from pentoses and hexoses degradation are furfural and 5-hydroxymethylfurfural (5-HMF) and, among phenolic compounds, 4-hydroxybenzoic acid (4-HBA) and vanillin should be highlighted among the most toxic ones (Tomás-Pejó et al., 2011). Here, we tested the enzymatic activity of Lfa2 toward pNPβGlu in the presence of different concentrations of furfural (0–1%), 5-HMF (0–1%), 4-HBA (0–2%) and vanillin (0–2%). Furfural and vanillin were not able to strongly inhibit Lfa2 activity, even at higher concentrations as 1 and 2%, Lfa2 activity has remained approximately 70 and 33%, respectively, when compared with the control, whereas in presence of 4-HBA 2%, Lfa2 activity was dramatically decreased. On the other hand, in the presence of higher concentrations of 5-HMF (1%), the Lfa2 activity was enhanced in more than 70%. Most cellulases are inhibited in the presence of this lignocellulose-derived compounds (Ximenes et al., 2010, 2011; Maruthamuthu and Van Elsas, 2017), however, cellulases are found to be more sensitive to inhibition by some phenolic compounds than β-glucosidases (Berlin et al., 2006; Ximenes et al., 2010). Furfural and 5-HMF concentrations in sugarcane bagasse hydrolysates pretreated by acid solution are 0.1–1.25% (w/v) and less than 0.1%, respectively (Chen et al., 2012; Tizazu and Moholkar, 2018), whereas in corn stover the levels of 5-HMF and furfural were 0.001–0.004% and 0.0026–0.02% (w/v), respectively (Du et al., 2010). Vanillin and 4-HBA appears in lower concentrations in corn stover hydrolysates, being vanillin ranging from 2.8 to 4.0 mg/L and 4-HBA around 0.028 mg/L. Interestingly, Lfa2 has remained active in higher concentrations of lignocellulose-derived compounds than the ones reported for sugarcane bagasse and corn stover hydrolysates. To the best of our knowledge, Lfa2 is the first β-glucosidase described in the literature as stimulated in presence of 5-HMF. Until now, two native β-glucosidases from the fungus *Clavispora* have been described as resistant to 5-HMF, but its activity was not enhanced in presence of the compound. Wang et al. (2016) showed that 0.12% furfural and 5-HMF did not affect the activity of these two β-glucosidases.

During the process of enzymatic lignocellulose degradation, β-glucosidases play an important role hydrolyzing cello-oligosaccharides – such as cellobiose and cellotrioses – into glucose (Singhania et al., 2013). Cello-oligosaccharides are produced during the degradation of cellulose by cellulases such as endo-β-glucanases and cellobiohydrolases, thus, β-glucosidase activity is essential for efficient plant biomass saccharification process, since cello-oligosaccharides may inhibit the activity of endo-β-glucanases and cellobiohydrolases. Chauve et al. (2010) demonstrated that a β-glucosidase from *Aspergillus niger* may be used to achieve complete hydrolysis of oligosaccharides produced by endoglucanases. Other studies have shown that the addition of β-glucosidases in hydrolysis of some lignocellulosic matters improved hydrolysis in around 20% when compared with non-supplemented reactions (Han and Chen, 2008; Pallapolu et al., 2011; Zhang et al., 2017). Here, we demonstrated that β-glucosidase Lfa2 acts in synergy with endoglucanase *Bs*Cel5A of *B. subtilis* hydrolyzing CMC. We showed in a straightforward experiment that addition of Lfa2 increased in 60% the glucose release compared to the non-supplemented reaction (*Bs*Cel5A alone).

## Conclusion

In summary, we have found and characterized a novel ethanol- and 5-hydroxymethyl furfural-stimulated β-glucosidase recovered from a Brazilian Secondary Atlantic Forest soil metagenome. The combined properties of Lfa2, including its increased activity in the presence of ethanol; medium tolerance level to glucose; high tolerance to 5-HMF; low responsiveness to several metal ions; synergistic effect when coupled to endoglucanase activity and broad substrate specificity, supported this enzyme as a very promising candidate for utilization in a wide range of industrial applications, such as cellulosic biomass degradation or flavor enhancement in winemaking and grape processing.

## Data Availability

Nucleotide sequence obtained for the plasmid insert have been deposited in the GenBank database under the accession number (MH397474), as described in Results session.

## Author Contributions

LA and M-EG designed the experiments. LA, LM, RS, and CW performed the experiments. LA and LM analyzed the data. LA and M-EG wrote the manuscript. All authors reviewed the manuscript.

## Conflict of Interest Statement

The authors declare that the research was conducted in the absence of any commercial or financial relationships that could be construed as a potential conflict of interest.

## Footnotes

1 https://web.expasy.org/translate/

2 http://pfam.xfam.org/

3 http://www.cbs.dtu.dk/services/SignalP/

4 https://web.expasy.org/protparam/

5 https://swissmodel.expasy.org/

6 https://zhanglab.ccmb.med.umich.edu/I-TASSER/

7 https://pymol.org/2/

8 https://www.ebi.ac.uk/Tools/msa/clustalo/

9 https://embnet.vital-it.ch/software/BOX_faq.html

10 http://www.cazy.org/

11 https://web.expasy.org/protparam/
