# Supplementary material for: Novel Ethanol- and 5-Hydroxymethyl Furfural-Stimulated β-Glucosidase Retrieved From a Brazilian Secondary Atlantic Forest Soil Metagenome
Source: Front Microbiol. 2018 Oct 29;9:2556. doi: 10.3389/fmicb.2018.02556 (PMC6215845; doi:10.3389/fmicb.2018.02556)
Supplement: Supplementary file 1 [file Data_Sheet_1.pdf]

Supplementary Material

Novel ethanol-stimulated β-glucosidase retrieved from a Brazilian Secondary Atlantic Forest soil metagenome

Luana de Fátima Alves[1], Luana Parras Meleiro[2], Roberto do Nascimento Silva[1], Cauã Antunes Westmann[3] and María-Eugenia Guazzaroni[4*]

[1]Department of Biochemistry, FMRP, University of São Paulo, Ribeirão Preto, SP, Brazil

[2]Department of Chemistry, FFCLRP, University of São Paulo, SP, Brazil

[3]Department of Cellular and Molecular Biology, FMRP, University of São Paulo, Ribeirão Preto, SP, Brazil

[4]Department of Biology, FFCLRP, University of São Paulo, SP, Brazil

*Correspondence:

María-Eugenia Guazzaroni

meguazzaroni@ffclrp.usp.br

Supplementary Figures

```
(3ABZ)          1 -------------------------------MSKFDVEQLLSELNQDEKISLLSAV------
(AGH13475.1)    1 MRKSIHQISLVILAVVAMCGYSLRMKAQQLKAENIDEVVKAMTLEEKCHMVLGRGMHF--
(Lfa2)          1 MKSRLVA-SLAVLLSLVVQRPLAQTPAPQLGKNTIAEVVAAMTTEEKAKLLVGMGMDLGI
(2X41)          1 ---------------------------------MEKVNEILSQLTLEEKVKLVVGVGLPGLF
(AGH13484.1)    1 MKKIML------LSATVL-ALAGCKGDPQLGKDSVDKVLKAMTLEEKVHFVIGTGMAGFG
(AGH13451.1)    1 MKKLLT------ICFVATTFGMTMNAQPKLSADNIDEVLKAMTLEEKAKLLVGGAN-NFF

(3ABZ)         26 --------------------DFWHTKKIERLGIPAVRVSDGPNGIRGTKFFDGV-----PS
(AGH13475.1)   59 -----------NDEAKFPGTAGSTFSIARLGVPETYCADSQQGLRMNATRAWD-HNDYYP
(Lfa2)         60 PGVSLISEDDQKIPEKVPGAAGRTHAIPRLGIPSLTLSDGPAGIRISPTRNGNTSKTYYA
(2X41)         30 G----------NPHSRVAGAAGETHPVPRVGLPAFVLADGPAGLRINPTRENDENTYYT
```

```
(AGH13484.1)   54 GE----SAVVGATKNIVPGAAGTTYPIERLGIPSIVLADGPAGLRIDPTREGD-ENTYYC
(AGH13451.1)   54 SA----NAVVGGEATLVAGAAGTTPEIARLGIPATVLTDGPAGVRINPTRRGT-DQTFYA

(3ABZ)         62 GCFPNGTGLASTFDRDLLETAGKLMAKESIAKNAAVILGPTTNMQRGPLGGRGFESFSED
(AGH13475.1)  107 TDFVASMTLASTWDREAAFKVGQGIGNEVREFGLDWILSPAMNLIRNPLCGRNHEYYSED
(Lfa2)        120 TGFPIATLVASSWDTNLANRVGTAMGSEARDYGVDILLAPAMNIHRNPLGGRNFEYYSED
(2X41)         79 TAFPVEIMLASTWNRELLEEVGKAMGEEVREYGVDVLLAPAMNIHRNPLCGRNFEYYSED
(AGH13484.1)  109 THFPIGTLLASTWNQELVESVGKAMGEEVHEYGADVYLAPALNIHRHPLNGRNFEYYSED
(AGH13451.1)  109 TAFPIGSCLASTWNTELVGQVGQAIGNETKEYRCDVILGPGMNLHRNPLCGRNFEYYSED

(3ABZ)        122 PYLAGMATSSVVKGMQGEGIAATVKHFVCNDLEDQRFSSNSIVSERALREIYLEPFRLAV
(AGH13475.1)  167 PYLSGTIAAGYVNGVQSEGTAACPKHFVANNQETNRNNNVSQVSQRALREIYLKAFEIMV
(Lfa2)        180 PLLSGKMAAVLCVGYSRMESEPPSNTSRQTTQEFNRMQSDSVVSERALREIYLKGLEIAV
(2X41)        139 PVLSGEMASSFVKGVQSQGVGACIKHFVANNQETNRMVVDTIVSERALREIYLRGFEIAV
(AGH13484.1)  169 PVVAGKTAAAYVRGVQSNDVGTSIKHFAYNNQETNRTGNNAVISPRAQREIYLKGFEITV
(AGH13451.1)  169 PFLTGKIAAAYINGVQKEDVGVSAKHFAVNSQETDRTSVDERLSQRAAREIYLRGFEIAV

(3ABZ)        182 KHANPVCIMTAYNKVNGEHCSQSKKLLIDILRDEWKWDGMLMSDWFGTYTTAA---AIKN
(AGH13475.1)  227 KESNPWTIMTSYNKLNGPYAVQNHDLLTTIVRNEWGWKGMYVSDWNAGDDAVA---AMLA
(Lfa2)        240 KESQPWTVMSSYNLINGTYASHSADLLTTLLRGEWGFKGFVMTDWFGGSNVIA---QMKA
(2X41)        199 KKSKPWSVMSAYNKLNGKYCSQNEWLLKKVLREEWGFEGFVMSDWYAGDNPVE---QLKA
(AGH13484.1)  229 KESDPWTVMSSYNKINGTYTSQSRDLITTVLRDEWGFKGLVMTDWFGGDNGAE---QIAA
(AGH13451.1)  229 RESNPWTIMASYNIINGTHAMGNRDLLTSILRDDWGYKGIVMTDWIGIRQGLTTISEVQA

(3ABZ)        239 GLDIEFPGPTRWRTRA---LVSHSL--NSREQITTEDVDDRVRQVLKMIKFVVDNLEKTG
(AGH13475.1)  284 GNDMLQPGQDKQ---------YQAILEAAQSGKLPMEVIDANVKRILEYV---VKT----H
(Lfa2)        297 GNEMIMPGVPPQ---------VPEIVNAVNTGTLSKEVLDQNVTRVLNII---LHA----P
(2X41)        256 GNDLIMPGKAYQVNTERRDEIEEIMEALKEGKLSEEVLDECVRNILKVL---VNA----P
(AGH13484.1)  286 GNDMLQPGTDLQ---------YQQIMDAIKDGSLSEDELNICVRRCLELV---ARS----P
(AGH13451.1)  289 GNDLMEPGQPAQ---------VQEIIDGVKNGKLAIADVDRNVRRMLEYI---VKT----P

(3ABZ)        294 IVENGPESTSNNTKETSDLLRKIAADSIVLLKNKNNILPLKKEDNIIVIGPNAKAKTSSG
(AGH13475.1)  329 NFKGYKYNNEPDLKAHAKVVREVGADGIVLLKNS-GVLPLVG-KRVALFGCTSYDWISGG
(Lfa2)        342 TFKGLKYSDQADLKTDAEIARETATEGMVLLKNDNKALPLAAPVKIGLFGISAYDLTAGG
(2X41)        309 SFKNYRYSNKPDLEKHAKVAYEAGAEGVVLLRNE-EALPLSENSKIALFGTGQIETIKGG
(AGH13484.1)  331 KAKGYAYSNKPDLKAHAAVTRQSALEGMVLLENN-GVLPLKDVKNVAVFGCTSFDFIAGG
(AGH13451.1)  334 SFRKYPASNKPDFTAHAAITRQSANEGIVLLKNN-GTLPWKNIKSVALFGENSYDFLSGG

(3ABZ)        354 GGSASM-NSYYVVSPYEGIVNKLGKEVDYTVGAYSHKSIGGLAESSLIDAAKPADAENSG
(AGH13475.1)  387 SGFGGTSVGHYTVSLIEGMRSV-GYEIYKPLIA---------------------------
(Lfa2)        402 TGSGDV-NKKYVVSLDQGLAQA-GFGLDQTLKT---------------------------
(2X41)        368 TGSGDT-HPRYAISILEGIKER-GLNFDEELAK---------------------------
(AGH13484.1)  390 TGSGNV-NRAYTVSLLDGLKNA-GFNVDESNKE---------------------------
(AGH13451.1)  393 TGSGCV-HPPYVVDMLEGLKNA-GIKSSETLTD---------------------------

(3ABZ)        413 LIAKFYSNPVEERSDDEEPFHVTKVNRSNVHLFDFKHEKVDPKNPYFFVTLTGQYVPQED
(AGH13475.1)  419 ----AYTQHIAAE---EKR---------------LFPNGRP------AFSLMPPARAD--
(Lfa2)        433 ----TYTDYLVQQ---KAT---------------RK-----RPGPFMPF-----PPIP--
(2X41)        399 ----TYEDYIKKM---RET---------------EEYK--PRRDSW---GTIIKPKLP--
(AGH13484.1)  421 ----ATLKHIADE---DAA---------------FKASLTDQLSAFYPT-----PRPS--
```

```
(AGH13451.1)  424 ----IYRKYIAYA---RVK---------------FQAERH--PAKWFQTEAMGQQKYP--

(3ABZ)        473 GDYIFSLQVYGSGLFYLNDELIIDQKHNQERGSFCFGAGTKERTKKLTLKKGQVYNVRVE
(AGH13475.1)  449 -EKQF-----------TADE----------------------------------------
(Lfa2)        459 -EMPV-----------AAER----------------------------------------
(2X41)        430 -ENFL-----------SEKE----------------------------------------
(AGH13484.1)  452 -ELVP-----------SAAE----------------------------------------
(AGH13451.1)  458 -EISL-----------SPIA----------------------------------------

(3ABZ)        533 YGSGPTSGLVGEFGAGGFQAGVIKAIDDDEEIRNAAELAAKHDKAVLIIGLNGEWETEGY
(AGH13475.1)  457 ------------------------------------LNAAIEGSDVAIISLG---RKSGEAA
(Lfa2)        467 ------------------------------------IAQMANQMDLAVITLG---RSSGEFV
(2X41)        438 ------------------------------------IHKLAKKNDVAVIVIS---RISGEGY
(AGH13484.1)  460 ------------------------------------LAASARANDVAIITFG---RNSGEFF
(AGH13451.1)  466 ------------------------------------VEKEVRGADAAIITIG---RQAGEGI

(3ABZ)        593 DRE----NMDLPKRTNELVRAVL----K-ANPNTVIVNQSGTPVEFPWLEDANALVQAWY
(AGH13475.1)  480 DRS--ESDFYLKDGEAQLIKAVSDAYHAKGKQVVVLLDICSPIDVASWQNQVDALVCTWQ
(Lfa2)        490 DRV-VENDFTVTASERGLVESVTSAFQAKGKKVVVVLNVGGPIEMASWRTLPDAVLLAWQ
(2X41)        461 DRKPVKGDFYLSDDETDLIKTVSREFHEQGKKVIVLLNIGSPVEVVSWRDLVDGILLVWQ
(AGH13484.1)  483 DRT--SADFSLSAKEKELLAKVTAAFHAKGKKVVVVLNVGGVIETASWKSVPDAILLAWQ
(AGH13451.1)  489 DRD-IETEFNLIPEERQLIFDVCQAFHAAGKPVVVIINSGSVIETASWSGYPDAILCAWQ

(3ABZ)        644 GGNELGNAIADVLYGDVVPNGKLSLSWPFKLQDNPAFLNFKTEF----------------
(AGH13475.1)  538 GGQESGFSVADVLSGKVNPSGKLPMTFQIKYGDAYADKNFPANVDDKTLGAMFMWGYDKD
(Lfa2)        549 PGQEGGNAITDVLTGKVNPSGRLPMTFPIAYGGMSHRPNVSRKS----FSRQTKHG----
(2X41)        521 AGQETGRIVADVLTGRINPSGKLPTTFPRDYSDVPS-WTFPGEP----------------
(AGH13484.1)  541 AGQEGGNSVTDILTGAKSPSGKLPMTFPVNLMDHASSANFPIDSDTGVYFTNRRED----
(AGH13451.1)  548 PGMEGGNSIADLLTGKVNPSGKLTMTWPIAATDHASTKNFPGALDDYSLMQMLGNG----

(3ABZ)        688 ------------GRVIYGEDIFVGYRYYEKLQRKVAFPFGYGLSYTTFELDISDFKVTD-
(AGH13475.1)  598 NTPKERQLQANIDFTNYEEDIYVGYRYFDSFSKPVAYPFGFGLSYTTFAYENLSVSETD-
(Lfa2)        601 ---LPTRLLVSHLKPFTTRGIYVGYRYYNTFGVKTAYDFGYGLSYTKFSYGNLKLSSNTF
(2X41)        564 ---------KDNPQKVVYEEDIYVGYRYYDTFGVEPAYEFGYGLSYTTFEYSDLNVSFDG-
(AGH13484.1)  597 ---VG---QKDVDVTKYEEGIYVGYRWFDKQNLPVSYPFGYGLSYTTFEYSAPAVANDG-
(AGH13451.1)  604 ---QP---IPGHAYTNHEEDIYVGYRFFDTFKRDVAYPFGYGLSYTTFEYSKPVVKAKG-

(3ABZ)        735 -DKIAISVDVKNTGDKFAGSEVVQVYFSALNS-KVSRPVKELKGFEKVH-LEPGEKKTVN
(AGH13475.1)  657 -GVFTVKVDVKNTGNK-AGRNVVELFVAAPNSKQLNKPEKELRNYAKTSLLKPGQTETVI
(Lfa2)        658 AGKVTATISVTNAGDV-AGKEVVQLYLGAPRQ-KLSKPERELRGFAKTRLLKPGESETLT
(2X41)        615 -ETLRVQYRIENTGGR-AGKEVSQVYIKAPKG-KIDKPFQELKAFHKTRLLNPGESEEVV
(AGH13484.1)  650 -KTVTAKVTVKNTGSV-AGKEAVQLYVSAPAG-TLDKPVKELKAYGKTKELAPGESQELT
(AGH13451.1)  657 -QTVEVSITVKNSGSV-SGKEVAQVYVAAPKG-RLEKPAQELKAFAKTRELQPGESQTLT

(3ABZ)        792 IDLELKDAISYFNEELGKWHVEAGEYLVSVGTSSDDILSVKEFKVEKELYWKGL------
(AGH13475.1)  715 MLVKTEDL-ASFNEKASAWKTDAGVYTFMICSSANQIEVQTSAKVKAWTKKV--NNVMKP
(Lfa2)        716 FTIAPQDL-ASFDSSSSSWVAEAGSYNVMVGASIQDIKTTATFKLASDLTVSKSQKLLAP
(2X41)        672 LEIPVRDL-ASFNG--EEWVVEAGEYEVRVGASSRNIKLKGTFSVGEERR-------FKP
(AGH13484.1)  707 LTFPTSEL-ASFDEEASAWKVDAGTYTFQFGASSRDVRCTATAEAAASETPA--NRVL--
(AGH13451.1)  714 MTIPVRNL-ASFDEANSQWLTEAGTYTFRIGANSRDIRCTAEAKLAEYTEQT--SNALAP
```

```
(3ABZ)                -----------
(AGH13475.1)   772 KLKLNLLKR--
(Lfa2)         775 TVTINELSSSK
(2X41)         722 -----------
(AGH13484.1)   762 -----LMK---
(AGH13451.1)   771 KQKLNLLKQ--
```

Figure S1. Full alignment of the amino acid sequences of β-glucosidase Lfa2 with other GH3 family β-glucosidases. Amino acid sequence alignment was carried out using the Clustal Omega server. Conserved residues are shaded using BOXSHADE (black background=strictly conserved; grey or white background=conservatively substituted or non-conserved). Asterisks indicate the nucleophile residue D283 and the acid/base amino acid residue E487. Full species names and Genbank, PDB or NCBI IDs of the amino acid sequences shown are as follows: *Kluyveromyces marxianus* PDB ID: 3ABZ (3ABZ); metagenomic β-glucosidases from cow rumen NCBI accession numbers: JX163905, JX163906 and JX163904 (AGH13475.1, AGH13484.1 and AGH13451.1); *Thermotoga neapolitana* PDB ID: 2X41 (2X41); and metagenomic β-glucosidase Lfa2 from a Secondary Atlantic Forest soil (this study) Genbank accession number: MH397474 (Lfa2).

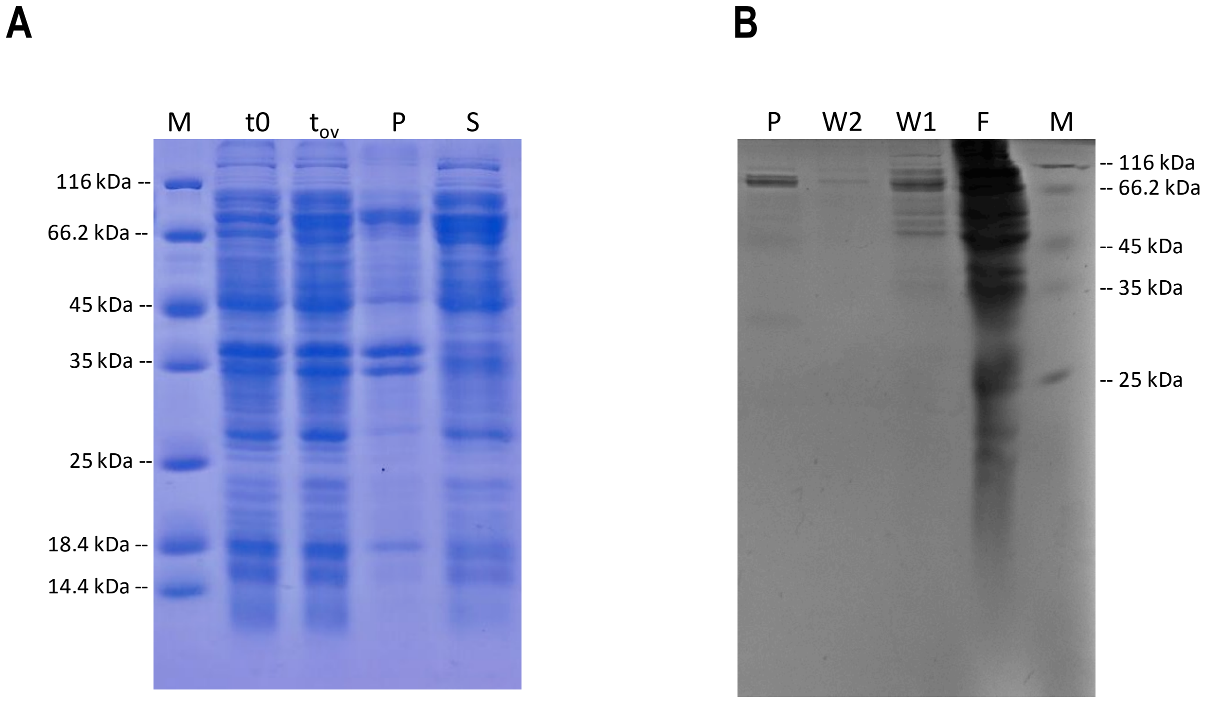

Figure S2. Overexpression and purification of recombinant Lfa2 in *Escherichia coli* DE3. (A) 12% SDS-PAGE analysis showing Lfa2 overexpression at 18°C. M, Marker; t0 and t_{ov} indicate times (0 and overnight expression - around 18 hours -, respectively) after 0.1 mM IPTG addition. P, pellet after cells sonication and centrifugation; S, supernatant after cells sonication and centrifugation. **(B)** 12% SDS-PAGE analysis showing the purification of recombinant Lfa2 by Ni^{2+} affinity

chromatography. M, Marker; F, whole cell content after loading; W1 and W2, washing through with 40 mM and 80 mM imidazole, respectively; P, purified eluate with 300 mM imidazole. As expected, purified recombinant Lfa2 (predicted molecular mass of 84 kDa) presents a molecular mass of between 66.2 kDa and 116 kDa.

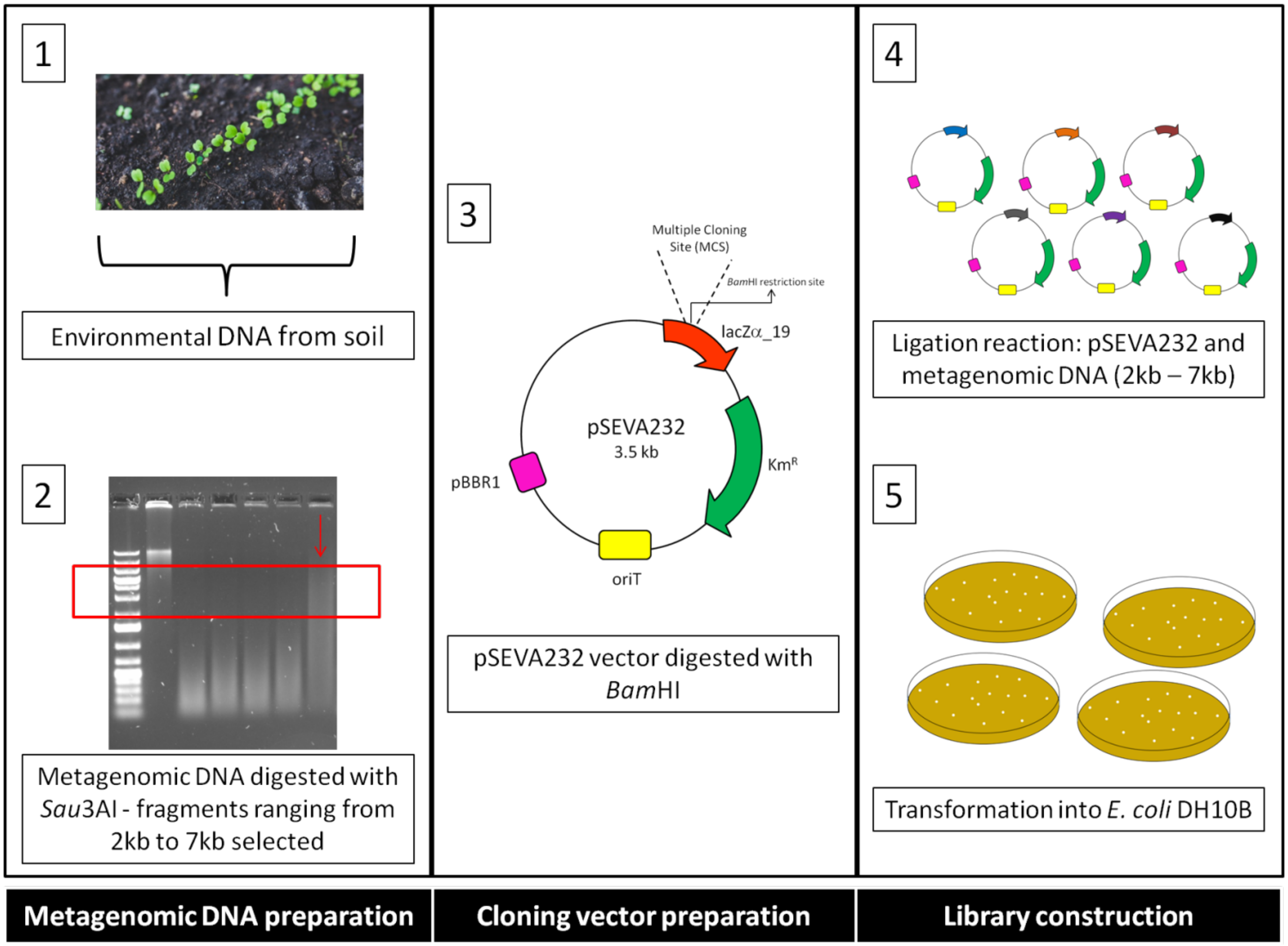

Figure S3. Library generation workflow. Firstly, 1) we have collected DNA from a Secondary Atlantic Forest region soil and the metagenomic DNA was extracted using the UltraClean™ Soil DNA isolation kit (Mo Bio, EUA), after 2) the metagenomic DNA was partially digested using *Sau*3AI (by combining enzyme concentration and time of reaction) in order to generate fragments of different sizes, once the restriction site for *Sau*3AI contain 4 nucleotides, it cuts the DNA more frequently than other enzymes with 6 nucleotides in the restriction site, for example. The metagenomic DNA fragments ranging from 2 kb to 7 kb were collected directly from an agarose gel and purified. 3) The plasmid vector pSEVA232 was digested in a unique site using *Bam*HI restriction enzyme (since the *Bam*HI restriction site is present in the multiple cloning site) and dephosphorylated by a Calf Intestinal Phosphatase. 4) Since *Bam*HI and *Sau*3AI present compatible cohesive ends, the library was constructed by a ligation reaction between the *Bam*HI-degested pSEVA232 vector and the *Sau*3AI-digested metagenomic DNA (selected fragments from 2 kb to 7 kb). 5) The ligation

reaction was transformed by electroporation in E. coli DH10B cells until obtain approximately 100,000 clones.

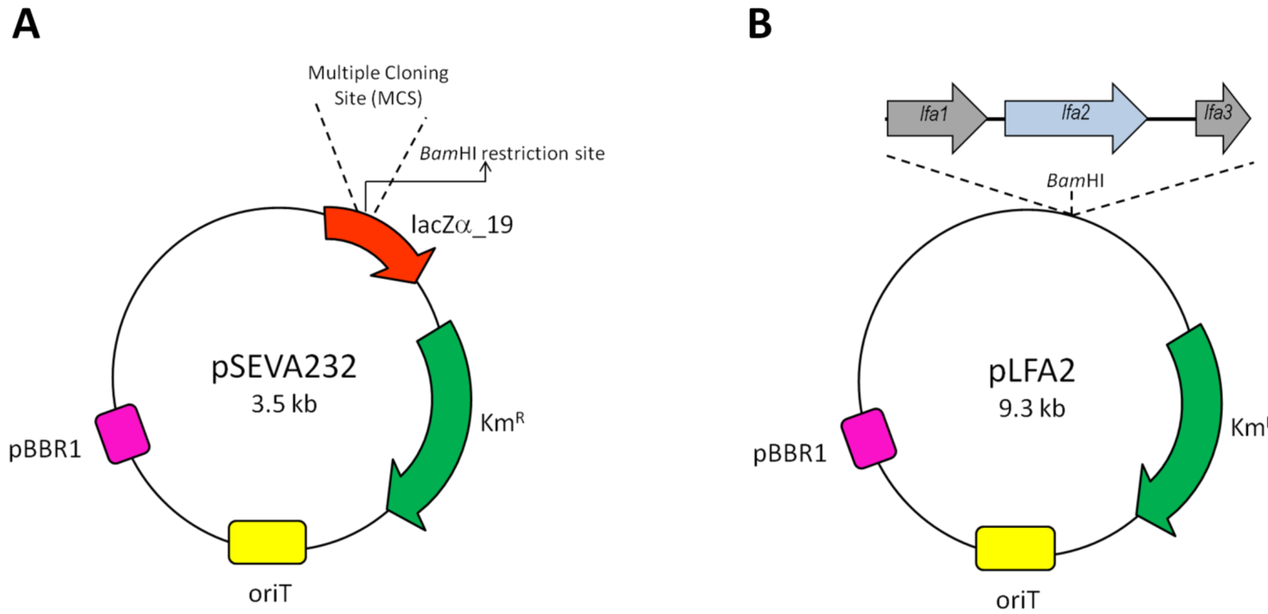

Figure S4. Plasmid maps of pSEVA232 and pLFA2. A) pSEVA232 plasmid vector (Silva-Rocha et al., 2013) present an approximate size of 3.5 kb and contain a wide host range origin of replication (pBBR1) which allows its replication in a large number of gram-negative bacteria. The vector also contain a origin of transfer (oriT) that allows the transference of the DNA that contains it from a bacterial host to a recipient cell during bacterial conjugation, in combination with a kanamycin resistance marker (Km^R) and a reporter gene lacZα (lacZα_19), which is used as a marker gene in white/ blue colony identification. In the figure, the *Bam*HI restriction site is highlighted. B) The pLFA2 plasmid was generated from the pSEVA232 backbone with the insertion of the metagenomic DNA from soil containing the lfa1, lfa2 and lfa3 ORFs in the *Bam*HI restriction site, yielding a plasmid of approximately 9.3 kb.

Supplementary tables

Table S1. Comparison of ethanol-tolerance of β-glucosidase Lfa2 and β-glucosidases from other microorganisms

β-glucosidase source	Remain activity/Ethanol concentration	Ethanol stimulation/Ethanol concentration	References
metagenomic	59% acitivity in 25% of ethanol (v/v)	1.7-fold in 10% ethanol (v/v)	this work
Thermoascus aurantiacus	not reported	1.3-fold in 30% ethanol (v/v)	(Parry et al., 2001)
Myceliophthora thermophila	Remains stable after 6 h of incubation in ethanol concentrations up to 50% (v/v) at 30°C.	2.4-fold in 15% ethanol (v/v)	(Karnaouri et al., 2013)
marine microbial metagenome (semi-rational engineered)	35% activity in 30% of ethanol (v/v)	not present	(Fang et al., 2016)
Lichtheimia ramosa	20% activity in 15% of ethanol (v/v)	1.2-fold in 5% ethanol (v/v)	(Garcia et al., 2015)
Bacillus cellulosilyticus	20% activity in 25% of ethanol (v/v)	not present	(Wu et al., 2018a)
marine Bacillus subtilis	70% activity in 14% of ethanol (v/v)	1.1-fold in 6% of ethanol (v/v)	(Baffi et al., 2013)
Sporidiobolus pararoseus	15% activity in 20% of ethanol (v/v)	1.5-fold in 5% of ethanol (v/v)	(Xue et al., 2018)
Thermobifida fusca	75% activity in 15% (v/v) (120 g/L)	not present	(Gosset and Martinez, 2012)
Candida molischiana 35M5N	100% acitivty in 11.5% (v/v) (2 M)	1.35-fold up to 5.8% (v/v) (1 M)	(Gueguen et al., 1996)

Table S2. Comparison of kinetic parameters of β-glucosidase Lfa2 and β-glucosidases from other sources

General β-glucosidases from bacteria, fungi and metagenomic studies			
β-glucosidase source	k_{cat}/K_M (*p*npβGlu)	k_{cat}/K_M (cellobiose)	References
Soil microbial metagenome	17.4 x 10^3 s^{-1} M^{-1} or 17.37 s^{-1} mM^{-1}	3.02 x 10^2 s^{-1} M^{-1} or 0.3 s^{-1} mM^{-1}	This work
Thermotoga petrophila	30800 s^{-1} mM^{-1}	NR[a]	(Haq et al., 2012)
Micrococcus antarcticus	1.12 x 10^3 s^{-1} mM^{-1}	NR[a]	(Fan et al., 2011)
Dictyoglomus turgidum	10400 s^{-1} mM^{-1}	NR[a]	(Kim et al., 2011)
Marinomonas MWYL1	500.5 s^{-1} mM^{-1}	395.8 $s^{-1}$$mM^{-1}$	(Zhao et al., 2012)
Bacillus halodurans	0.187 s^{-1} mM^{-1}	NR[a]	(Naz et al., 2010)
Sulfolobus solfataricus	0.09 s^{-1} mM^{-1}	NR[a]	(Cobucci-Ponzano et al., 2010)
Phoma sp.	1.6 s^{-1} M^{-1}	215.8 × 10^5 $s^{-1}$$M^{-1}$	(Choi et al., 2011)
Daldinia eschscholzii	2.27 × 10^3 s^{-1} M^{-1}	NR[a]	(Karnchanatat et al., 2007)
Fomitopsis pinicola	1700 s^{-1} mM^{-1}	NR[a]	(Joo et al., 2009)
Cow rumen metagenome	SRF2g14 - 10.76 × 10^3 s^{-1} M^{-1}, LAB25g2 - 9.20 × 10^2 s^{-1} M^{-1}, SRF2g18 - 1.59 × 10^2 s^{-1} M^{-1}, LAB20g4 - 29.49 × 10^3 s^{-1} M^{-1}	SRF2g14 - 47.64 s^{-1} M^{-1}, LAB25g2 - 20.84 s^{-1} M^{-1}, SRF2g18 - 3.35 s^{-1} M^{-1}, LAB20g4 - 28.75 s^{-1} M^{-1}	(Pozo et al., 2012)
Yak rumen metagenome	11900 s^{-1} M^{-1}	NR[a]	(Bao et al., 2012)

Faecal samples from earthworm species	G05.26 - 2.9×10^4 s^{-1} M^{-1}, G05.27 - 1.5×10^3 s^{-1} M^{-1}, G06.24 - 5.5×10^5 s^{-1} M^{-1}, G07.33 - 0.8×10^3 s^{-1} M^{-1}, G08.17 - 1.2×10^3 s^{-1} M^{-1}, G10.10 - 3.9×10^3 s^{-1} M^{-1}	NR[a]	(Beloqui et al., 2010)
Amazonian water sample	6s^{-1} mM^{-1}	0.6 s^{-1} mM^{-1}	(Toyama et al., 2018)
Bacillus cellulosilyticus	70.3 s^{-1}	45.8 s^{-1}	(Wu et al., 2018b)
Talaromyces amestolkiae	9710.2 mM^{-1} s^{-1}	3308.4 mM^{-1} s^{-1}	(Méndez-Líter et al., 2018)
Saccharophagus degradans	NR[a]	6.5×10^{-1}	(Kim et al., 2018)
Commercial β-glucosidases			
Aspergillus niger cellulase powder (Sigma Aldrich, USA)	1,33 x 10^8 s^{-1} mM^{-1}	8.3 x 10^7 s^{-1} mM^{-1}	(Seidle et al., 2004)
Novozymes SP188 (Novozymes, Denmark)	46 s^{-1} mM^{-1}	35.92 s^{-1} mM^{-1}	(Chauve et al., 2010)

[a] NR: data not reported

References

Baffi, M. A., Martin, N., Tobal, T. M., Ferrarezi, A. L., Lago, J. H. G., Boscolo, M., et al. (2013). Purification and characterization of an ethanol-tolerant β-glucosidase from sporidiobolus pararoseus and its potential for hydrolysis of wine aroma precursors. *Appl. Biochem. Biotechnol.* 171, 1681–1691. doi:10.1007/s12010-013-0471-0.

Bao, L., Huang, Q., Chang, L., Sun, Q., Zhou, J., and Lu, H. (2012). Cloning and characterization of two β-glucosidase/xylosidase enzymes from yak rumen metagenome. *Appl. Biochem. Biotechnol.* 166, 72–86. doi:10.1007/s12010-011-9405-x.

Beloqui, A., Nechitaylo, T. Y., López-Cortés, N., Ghazi, A., Guazzaroni, M. E., Polaina, J., et al. (2010). Diversity of glycosyl hydrolases from cellulose-depleting communities enriched from casts of two earthworm species. *Appl. Environ. Microbiol.* 76, 5934–5946. doi:10.1128/AEM.00902-10.

Chauve, M., Mathis, H., Huc, D., Casanave, D., Monot, F., and Ferreira, N. L. (2010). Comparative kinetic analysis of two fungal β-glucosidases. *Biotechnol. Biofuels* 3, 1–8. doi:10.1186/1754-6834-3-3.

Choi, J. Y., Park, A. R., Kim, Y. J., Kim, J. J., Cha, C. J., and Yoon, J. J. (2011). Purification and characterization of an extracellular β-glucosidase produced by phoma sp. KCTC11825BP isolated from rotten mandarin peel. *J. Microbiol. Biotechnol.* 21, 503–508. doi:10.4014/jmb.1102.02014.

Cobucci-Ponzano, B., Aurilia, V., Riccio, G., Henrissat, B., Coutinho, P. M., Strazzulli, A., et al. (2010). A new archaeal β-glycosidase from Sulfolobus solfataricus: Seeding a novel retaining β-glycan-specific glycoside hydrolase family along with the human non-lysosomal glucosylceramidase GBA. *J. Biol. Chem.* 285, 20691–20703. doi:10.1074/jbc.M109.086470.

Fan, H. X., Miao, L. L., Liu, Y., Liu, H. C., and Liu, Z. P. (2011). Gene cloning and characterization of a cold-adapted β-glucosidase belonging to glycosyl hydrolase family 1 from a psychrotolerant bacterium Micrococcus antarcticus. *Enzyme Microb. Technol.* 49, 94–99. doi:10.1016/j.enzmictec.2011.03.001.

Fang, W., Yang, Y., Zhang, X., Yin, Q., Zhang, X., Wang, X., et al. (2016). Improve ethanol tolerance of β-glucosidase Bgl1A by semi-rational engineering for the hydrolysis of soybean isoflavone glycosides. *J. Biotechnol.* 227, 64–71. doi:10.1016/j.jbiotec.2016.04.022.

Garcia, N. F. L., da Silva Santos, F. R., Gonçalves, F. A., da Paz, M. F., Fonseca, G. G., and Leite, R. S. R. (2015). Production of β-glucosidase on solid-state fermentation by Lichtheimia ramosa in agroindustrial residues: Characterization and catalytic properties of the enzymatic extract. *Electron. J. Biotechnol.* 18, 1–6. doi:10.1016/j.ejbt.2015.05.007.

Gosset, G., and Martinez, A. (2012). Cell surface display of a b -glucosidase employing the type V secretion system on ethanologenic Escherichia coli for the fermentation of cellobiose to ethanol. 1141–1152. doi:10.1007/s10295-012-1122-0.

Gueguen, Y., Chemardin, P., Janbon, G., Arnaud, A., and Galzy, P. (1996). A Very Efficient β-Glucosidase Catalyst for the Hydrolysis of Flavor Precursors of Wines and Fruit Juices. *J. Agric. Food Chem.* 44, 2336–2340. doi:10.1021/jf950360j.

Haq, I. U., Khan, M. A., Muneer, B., Hussain, Z., Afzal, S., Majeed, S., et al. (2012). Cloning, characterization and molecular docking of a highly thermostable β-1,4-glucosidase from Thermotoga petrophila. *Biotechnol. Lett.* 34, 1703–1709. doi:10.1007/s10529-012-0953-0.

Joo, A. R., Jeya, M., Lee, K. M., Sim, W. Il, Kim, J. S., Kim, I. W., et al. (2009). Purification and characterization of a β-1,4-glucosidase from a newly isolated strain of Fomitopsis pinicola. *Appl. Microbiol. Biotechnol.* 83, 285–294. doi:10.1007/s00253-009-1861-7.

Karnaouri, A., Topakas, E., Paschos, T., Taouki, I., and Christakopoulos, P. (2013). Cloning, expression and characterization of an ethanol tolerant GH3 β-glucosidase from *Myceliophthora thermophila*. *PeerJ* 1, e46. doi:10.7717/peerj.46.

Karnchanatat, A., Petsom, A., Sangvanich, P., Piaphukiew, J., Whalley, A. J. S., Reynolds, C. D., et al. (2007). Purification and biochemical characterization of an extracellular β-glucosidase from the wood-decaying fungus Daldinia eschscholzii (Ehrenb.:Fr.) Rehm. *FEMS Microbiol. Lett.* 270, 162–170. doi:10.1111/j.1574-6968.2007.00662.x.

Kim, D. H., Kim, D. H., Lee, S. H., and Kim, K. H. (2018). A novel β-glucosidase from Saccharophagus degradans 2-40Tfor the efficient hydrolysis of laminarin from brown macroalgae. *Biotechnol. Biofuels* 11, 1–10. doi:10.1186/s13068-018-1059-2.

Kim, Y. S., Yeom, S. J., and Oh, D. K. (2011). Characterization of a GH3 family ??-glucosidase from Dictyoglomus turgidum and its application to the hydrolysis of isoflavone glycosides in spent coffee grounds. *J. Agric. Food Chem.* 59, 11812–11818. doi:10.1021/jf2025192.

Méndez-Líter, J. A., De Eugenio, L. I., Prieto, A., and Martínez, M. J. (2018). The β-glucosidase secreted by Talaromyces amestolkiae under carbon starvation: A versatile catalyst for biofuel production from plant and algal biomass. *Biotechnol. Biofuels* 11, 1–14. doi:10.1186/s13068-018-1125-9.

Naz, S., Ikram, N., Rajoka, M. I., Sadaf, S., and Akhtar, M. W. (2010). Enhanced production and characterization of a beta-glucosidase from Bacillus halodurans expressed in Escherichia coli. *Biochem. Biokhimii͡a* 75, 513–25. doi:BCM75049998 [pii].

Parry, N. J., Beever, D. E., Owen, E., Vandenberghe, I., Van Beeumen, J., and Bhat, M. K. (2001). Biochemical characterization and mechanism of action of a thermostable beta-glucosidase purified from Thermoascus aurantiacus. *Biochem. J.* 353, 117–127. doi:10.1042/0264-6021:3530117.

Pozo, M. V. Del, Fernández-arrojo, L., Gil-martínez, J., Montesinos, A., Chernikova, T. N., Nechitaylo, T. Y., et al. (2012). Microbial β -glucosidases from cow rumen metagenome enhance the saccharification of lignocellulose in combination with commercial cellulase cocktail. 1–13.

Seidle, H. F., Marten, I., Shoseyov, O., and Huber, R. E. (2004). Physical and kinetic properties of the family 3 β-glucosidase from Aspergillus niger which is important for cellulose breakdown. *Protein J.* 23, 11–23. doi:10.1023/B:JOPC.0000016254.58189.2a.

Silva-Rocha, R., Martínez-García, E., Calles, B., Chavarría, M., Arce-Rodríguez, A., De Las Heras, A., et al. (2013). The Standard European Vector Architecture (SEVA): A coherent platform for the analysis and deployment of complex prokaryotic phenotypes. *Nucleic Acids Res.* 41, 666–675. doi:10.1093/nar/gks1119.

Toyama, D., de Morais, M. A. B., Ramos, F. C., Zanphorlin, L. M., Tonoli, C. C. C., Balula, A. F., et al. (2018). A novel β-glucosidase isolated from the microbial metagenome of Lake Poraquê (Amazon, Brazil). *Biochim. Biophys. Acta - Proteins Proteomics*, 0–1. doi:10.1016/j.bbapap.2018.02.001.

Wu, J., Geng, A., Xie, R., Wang, H., and Sun, J. (2018a). Characterization of cold adapted and ethanol tolerant β-glucosidase from Bacillus cellulosilyticus and its application for directed hydrolysis of cellobiose to ethanol. *Int. J. Biol. Macromol.* 109, 872–879. doi:10.1016/j.ijbiomac.2017.11.072.

Wu, J., Geng, A., Xie, R., Wang, H., and Sun, J. (2018b). Characterization of cold adapted and ethanol tolerant β-glucosidase from Bacillus cellulosilyticus and its application for directed hydrolysis of cellobiose to ethanol. *Int. J. Biol. Macromol.* 109, 872–879. doi:10.1016/j.ijbiomac.2017.11.072.

Xue, D., Zeng, X., Gong, C., Lin, D., and Yao, S. (2018). A cold adapt and ethanol tolerant endoglucanase from a marine Bacillus subtilis. *Chinese J. Chem. Eng.* doi:10.1016/j.cjche.2018.02.007.

Zhao, W., Peng, R., Xiong, A., Fu, X., Tian, Y., and Yao, Q. (2012). Expression and characterization of a cold-active and xylose-stimulated β-glucosidase from Marinomonas MWYL1 in Escherichia coli. *Mol. Biol. Rep.* 39, 2937–2943. doi:10.1007/s11033-011-1055-0.
